# Effects of biochar amendment on the phytoextraction of twenty potentially toxic elements in fly ash contaminated soils

**DOI:** 10.3389/fpls.2026.1783879

**Published:** 2026-03-31

**Authors:** Santosh Rajbanshi, Maheteme Gebremedhin, James C. Hower, George Antonious, Suraj Upadhaya, Jacob Brown, Ife Familusi

**Affiliations:** 1 College of Agriculture, Health, and Natural Resources, Kentucky State University, Frankfort, KY, United States; 2 University of Kentucky Center for Applied Energy Research, Lexington, KY, United States; 3 Department of Earth & Environmental Sciences, University of Kentucky, Lexington, KY, United States

**Keywords:** potentially toxic elements, phytoextraction, biochar, fly ash, heavy metals

## Abstract

**Introduction:**

Coal Fly Ash (FA) poses a significant environmental challenge due to its physicochemical properties and its enrichment in potentially toxic elements (PTEs). Phytoextraction can be a cost-effective and eco-friendly approach to remediating sites contaminated with FA and PTEs. However, the role of soil amendments, such as biochar, in phytoextraction of PTEs from FA-contaminated soils remains underexplored.

**Methods:**

This study investigated the effects of biochar on plant biomass production and patterns of PTE accumulation in FA-amended soils using a controlled greenhouse experiment. Four plant species—switchgrass, tall fescue, hairy vetch, and sericea lespedeza—were grown in varying proportions of soil, FA, and biochar. Shoot elemental concentrations were quantified for 20 PTEs using ICP-AES and analyzed using correlation-based and multivariate analyses.

**Results and discussion:**

The results showed that FA amendment significantly reduced the plant shoot and root biomasses, while biochar enhanced the biomass accumulation of plants. Analysis of PTE bioaccumulation behavior of plant species revealed that Ca-Mg had a strong negative correlation with Ba-Sr uptake and very weak relationship with Al-Si. A weaker relationship between the bioaccumulation behavior of Al-Si and Fe-S was recorded in this study. Legumes (hairy vetch and sericea lespedeza) clustered towards Ca-Mg, while grasses (tall fescue and switchgrass) showed clustering towards Al-Si.

## 1 Introduction

Potentially Toxic Elements (PTEs) refer to any element that can cause biological or environmental effects, when present above certain threshold concentrations. They include both essential and non-essential elements capable of causing phytotoxic effects, for example, Fe, Mn, Mo, As, Hg, and Cd (Antoniadis et al., 2019; Thalassinos et al., 2023). The threshold concentration and phytotoxic effects largely depend on the type and characteristics of the element, plant species, and soil properties (Briffa et al., 2020). Common toxicity symptoms due to PTEs include reduction in the photosynthetic rates, N-metabolism inhibition, biomass reduction, nutrient uptake suppression, and chlorosis and necrosis (Emamverdian et al., 2015). Additionally, PTEs pose a significant threat to humans through food chain contamination (occurs through bioaccumulation and biomagnification in different trophic levels of the food chain). For example, Pb intoxication in children can result in severe neurodevelopmental impairments, including intellectual disability, memory loss, learning difficulties, and motor coordination challenges (WHO, 1997). Arsenic poisoning can manifest in a range of health issues, such as cardiovascular disease, neurological damage, and dermatological disorders, such as skin cancer (WHO, 1997). In addition, Cadmium accumulates in the kidneys, leading to chronic renal dysfunction (WHO, 1997). Mercury exposure, particularly during prenatal development, can cause severe neurological damage, characterized by tremors, muscle atrophy, visual impairment, and physical abnormalities (WHO, 1997).

PTEs have the potential to impact soil physical (Angon et al., 2024), chemical (Liu et al., 2005), and biological properties (Zhu et al., 2023) directly. They impact soil physical properties by disrupting soil structure (Alloway, 2013), altering soil texture (Angon et al., 2024), reducing soil porosity (Adriano, 2017), decreasing water holding capacity and increasing surface runoff (Imfeld et al., 2020), and weakening soil aggregate stability that ultimately increases soil erosion (Xie et al., 2016). Similarly, PTEs impact soil chemical properties through pH alteration (Hu et al., 2018), nutrient imbalance (Kabata-Pendias and Mukherjee, 2007), degradation of soil organic matter (Angon et al., 2024), influence on the redox reaction that alters the mobility and availability of nutrients (Alloway, 2013), and the adsorption and desorption processes, reducing bioavailability to the plants (Angon et al., 2024). Finally, they impact soil’s biological properties by inhibiting microbial growth and development, disrupting the cellular structures of the microbial population, and inhibiting the enzymatic activities in the soil (Bortoloti and Baron, 2022).

There are numerous sources of soil contamination by PTEs. Among them, industrialization is a significant source of contamination (Karthika et al., 2022). Fly ash (FA), a byproduct of coal combustion in coal-fired power plants, contains a range of these PTEs, such as Al, Fe, Si, Mg, Ti, As, and Ba, that contaminate soil and make it unfit for agricultural production (Electric Power Research Institute (EPRI), 2009). Over 500 million tons of FA are produced worldwide annually, of which only 25-30% is effectively reused (Sanalkumar et al., 2019). In the United States alone, the FA production in 2024 exceeded 24 Mt (ACAA, 2025). The continued large-scale generation of FA poses serious threat to the plant, soil, human, and environmental health. In addition, FA is often spread on the exposed land surfaces for its disposal, potentially leading to widespread accumulation and environmental harm (Abhishek et al., 2025).

The sites contaminated with FA require an economically and environmentally viable remediation approach. Traditional approaches like excavation, electrokinesis, soil washing, etc., are quicker approaches for site remediation, but lack viability in terms of environmental and economic perspectives (Ali et al., 2013; DalCorso et al., 2019; McGrath et al., 2002; Wei et al., 2008). Phytoextraction, the use of plant species that can thrive in the heavily contaminated sites with PTEs and can accumulate PTEs in their biomass without showing toxicity symptoms, is considered a suitable solution to this issue (Ali et al., 2013; Nepal et al., 2024). Switchgrass (*Panicum virgatum* L.) (Shrestha et al., 2019), tall fescue (*Festuca arundinacea*) (Babincev, 2017), hairy vetch (*Vicia villosa*) (Pinna et al., 2024), and sericea lespedeza (*Lespedeza cuneata*) (Chekol and Vough, 2002) are common plant species in marginalized lands and have shown promising results in phytoremediation. In addition, tall fescue and switchgrass can be used in biofuel generation, which is helpful in the safe disposal strategy for PTE-contaminated biomasses (Kanapeckas et al., 2011; Shen et al., 2013).

However, the success of phytoextraction largely depends on the plants’ growth and performance. One of the various approaches to optimizing plant performance is soil amendments, such as biochar. Biochar is a high-carbon charcoal-like substance, produced by pyrolysis, where plant biomass sources, such as plant residues, woodchips, etc., are heated at high temperature and pressure in the near or complete absence of oxygen (Sanchez and Belmont, 2025). Biochar improves soil properties, including soil carbon content, water-holding capacity and porosity, Cation Exchange Capacity (CEC), soil aggregate stability, and microbial activity, and reduces irrigation and fertilizer requirements (Li et al., 2017; Premalatha et al., 2023; Wei et al., 2023). It can play a crucial role in optimizing the growth and performance of plants used in phytoextraction of PTEs from FA and PTE-contaminated sites. Moreover, very limited studies have examined the effects of biochar on PTE mobilization and the phytoextraction potential of plants at FA-contaminated sites. Therefore, silty loam soils typically found at the Harold R. Research and Demonstration Farm, Frankfort, Kentucky, were amended with both FA and biochar in and the impacts of biochar on PTEs availability for plant bioaccumulation were determined. The specific questions we are investigating in this study are as follows:

- How do switchgrass, tall fescue, hairy vetch, and sericea lespedeza differ in their potential to accumulate PTEs in their shoots from FA contaminated sites?
- How does FA contamination in the soil affect the biomass yields of switchgrass, tall fescue, hairy vetch, and sericea lespedeza?
- Does the addition of biochar in the FA contaminated soils improve the bioaccumulation of the PTEs in plants?

## 2 Methodology

### 2.1 Experimental site

This study is a greenhouse-based experiment with a controlled temperature and humidity microclimate. It was conducted at the Kentucky State University’s Harold R. Benson Research and Demonstration Farm, located in the southern part of Franklin County, Kentucky.

### 2.2 Research design

This experiment was conducted using a Factorial Randomized Complete Block Design (RCBD) with two factors, viz., treatments and species. There were six treatments and four plant species, which were replicated four times, resulting in 96 pots used. Each treatment-species combination was blocked by replication. Within each block, treatment–species assignments to pot positions were randomized using a reproducible algorithm. A Completely Randomized Design (CRD) since it was a greenhouse experiment was considered, however, the distribution of sunlight varied across the greenhouse. Hence, to homogenize the distribution of the sun across replications, RCBD was selected. The error degree of freedom in this case was (r-1)(ts-1) = (4-1)(6x4-1) = 69, which was sufficient to detect moderate-to-significant effects while balancing precision with resource constraints.

### 2.3 Treatment and species details

For this study, Maury silt loam soil, the soil texture commonly found on this site, of approximately six inches (15.24 cm) soil depth was used for this research from one of the fields at the Harold R. Benson Farm. The biochar used in this study was commercially obtained from the American Biochar Company and was produced from southern yellow pine via pyrolysis. According to the manufacturer’s technical data sheet, the biochar was generated under high-temperature pyrolysis conditions (550–900 °C), which are characteristic of slow pyrolysis processes designed to maximize biochar yield. Detailed information on heating rate and residence time was not provided by the manufacturer. Finally, the fly ash used in our study was class F fly ash from Kentucky power plants that burned high-S, high volatile bituminous coal from the Illinois Basin (Hower et al., 1996, Hower et al., 2021; U. S. Geological Survey (USGS), 2002).

Six different combinations of soil, biochar, and fly ash were used in pots as treatments, with varying concentrations. A total of 3 kg of soil mix was filled in each pot. Those concentrations for each treatment are as follows:

1. Treatment 1 (T1): Control (3000 gm).
2. Treatment 2 (T2): 90% Soil (2700 gm) + 10% Fly Ash (300 gm).
3. Treatment 3 (T3): 90% Soil (2700 gm) + 10% Biochar (300 gm).
4. Treatment 4 (T4): 90% Soil (2700 gm) + 5% Biochar (150gm) + 5% Fly Ash (150 gm).
5. Treatment 5 (T5): 90% Soil (2700 gm) + 2.5% Fly Ash (75 gm) + 7.5% Biochar (225 gm).
6. Treatment 6 (T6): 90% Soil (2700gm) + 7.5% Fly Ash (225 gm) + 2.5% Biochar (75gm).

In addition to the treatments, four plant species were used in the study: switchgrass, hairy vetch, tall fescue, and sericea lespedeza. Among them, switchgrass and tall fescue are grass species, whereas hairy vetch and sericea lespedeza are legume species. They were grown for a period of three months (late April – late July, 2024), with the primary objective of identifying plant species that exhibit tolerance, enhanced germination success, and high bioaccumulation of PTEs in FA-contaminated soils.

#### 2.3.1 Switchgrass (*Panicum virgatum*)

It is a 1- 1.5-m tall, hardy, drought-resistant, perennial warm-season grass in the Poaceae family native to the United States except California and the Pacific Northwest (USDA & NRCS, 2011). It is widely used in soil conservation projects, phytoremediation projects, fiber production, heat and electricity generation, atmospheric CO_2_ sequestration, and as a biomass crop for ethanol and butanol (Wisconsin Horticulture - Division of Extension, n.d.).

#### 2.3.2 Hairy vetch (*Vicia villosa*)

It is a cool-season annual or biennial climber in the Fabaceae family growing up to 1.83 meters long. It is commonly used in nitrogen fixation in the soil as a cover crop and in soil conservation projects to prevent erosion due to its dense root system (Vandevender, 2002).

#### 2.3.3 Tall fescue (*Festuca arundinacea*)

It is a cool-season, hardy, resilient, and bushy grass of the Poaceae family growing at a height of around 46–92 cm at maturity. It is widely used in soil health enhancement projects, soil erosion control due to its dense growth habit, and in soil reclamation projects (Mohlenbrock, 1989).

#### 2.3.4 Sericea lespedeza (*Lespedeza cuneata*)

It is a perennial, warm-season perennial leguminous shrub, invasive to North America belonging to the Fabaceae family. It is widely used in soil erosion control projects, for nitrogen fixation in the soil, and soil reclamation projects (Cummings et al., 2017).

### 2.4 Data collection

Data collection was done in three phases for this experiment. First, the baseline concentrations of the 46 PTEs were collected from available soil, biochar, and FA samples, as shown in **Table 1**. Baseline chemical analysis of soil, fly ash (FA), and biochar samples involved multiple standardized digestion and analytical methods. Rare earth elements (Ce, Er, Yb, Eu, Y, Dy, Th, Nd, Gd, Ho, La, Lu, Pr, Sm, Sc, Tb, Tm) and U were quantified using aqua regia digestion coupled with EPA Method 6020. Mercury (Hg) was analyzed using EPA Method 7471A. Major elements (Al, Ca, Fe, Mg, Mn, Si, and Ti) were determined through borate fusion digestion (ASTM D4698, modified) followed by EPA Method 6010C. Finally, trace elements, including Sb, As, Ba, Be, Bi, B, Cd, Cr, Cu, Pb, Li, Mo, Ni, Se, Ag, Sr, S, Sn, V, and Zn, were analyzed using microwave digestion followed by EPA Method 6010C. The baseline results showed the presence of 36 PTEs in at least one of the soil, fly ash, and biochar samples. Among these, 25 elements exhibited higher concentrations in the fly ash compared to soil and biochar samples. Based on their relative abundance, 20 dominant elements (Al, As, B, Ba, Ca, Fe, Si, Ti, Mg, S, V, Cr, Zn, Mn, Ni, Cu, Li, Pb, Mo, and Sr) were considered for further evaluation in this study. Second, the dry biomass weights of the plants’ roots and shoots were recorded after their harvesting in the greenhouse. The biomass of the plant’s roots and shoots were air-dried for a week in the greenhouse, and then their weights were recorded on a weighing scale with a three-digit precision. Finally, the concentrations of PTEs in the plant’s shoots were analyzed. It included both vegetative parts, such as leaves and stems, and reproductive parts, such as flowers. The samples collected after the plants’ harvest were stored in zip lock bags in a refrigerator at 6 °C before their analysis for PTE concentration.

**Table 1:** Baseline concentration of all 46 PTEs in the soil, FA, and biochar samples.

PTEs	Concentration (mg/kg)		
	Soil	Fly ash	Biochar
Lithium (Li)	29.2	161	<24.4 (BDL)
Beryllium (Be)	<4.8 (BDL)	16.5	<4.9 (BDL)
Boron (B)	50.4	544	<24.4 (BDL)
Magnesium (Mg)	3980	5600	3110
Aluminum (Al)	48500	113000	978
Silicon (Si)	340000	199000	11400
Sulfur (S)	401	4230	153
Calcium (Ca)	4410	24500	10800
Scandium (Sc)	2.35	8.61	<0.99 (BDL)
Titanium (Ti)	5730	6330	<242 (BDL)
Vanadium (V)	61.5	533	<4.9 (BDL)
Chromium (Cr)	57.2	371	78.3
Manganese (Mn)	1730	311	1010
Iron (Fe)	29700	201000	1370
Nickel (Ni)	27	242	51.5
Copper (Cu)	0	177	15
Zinc (Zn)	66.9	354	<24.4 (BDL)
Arsenic (As)	<24.1 (BDL)	74.3	<24.4 (BDL)
Selenium (Se)	<24.1 (BDL)	<24.2 (BDL)	<24.4 (BDL)
Strontium (Sr)	63.2	65.8	35.1
Yttrium (Y)	20.6	20.6	<0.99 (BDL)
Molybdenum (Mo)	<9.7 (BDL)	83.8	<9.8 (BDL)
Silver (Ag)	<24.1 (BDL)	<24.2 (BDL)	<24.4 (BDL)
Cadmium (Cd)	<4.8 (BDL)	<4.8 (BDL)	<4.9 (BDL)
Tin (Sn)	<9.7 (BDL)	<9.7 (BDL)	37.1
Antimony (Sb)	<24.1 (BDL)	<24.2 (BDL)	<24.4 (BDL)
Barium (Ba)	161	118	47.3
Lanthanum (La)	17.3	12.1	<0.99 (BDL)
Cerium (Ce)	42.2	26.6	1.41
Praseodymium (Pr)	4.34	3.21	<0.99 (BDL)
Neodymium (Nd)	18	13.8	<0.99 (BDL)
Samarium (Sm)	3.67	3.47	<0.99 (BDL)
Europium (Eu)	<0.99 (BDL)	<0.99 (BDL)	<0.99 (BDL)
Gadolinium (Gd)	3.68	4.45	<0.99 (BDL)
Terbium (Tb)	<0.99 (BDL)	<0.99 (BDL)	<0.99 (BDL)
Dysprosium (Dy)	3.06	4.08	<0.99 (BDL)
Holmium (Ho)	<0.99 (BDL)	<0.99 (BDL)	<0.99 (BDL)
Erbium (Er)	1.66	2.17	<0.99 (BDL)
Thulium (Tm)	<0.99 (BDL)	<0.99 (BDL)	<0.99 (BDL)
Ytterbium (Yb)	1.25	1.92	<0.99 (BDL)
Lutetium (Lu)	<0.99 (BDL)	<0.99 (BDL)	<0.99 (BDL)
Mercury (Hg)	0.0337	<0.03 (BDL)	<0.03 (BDL)
Lead (Pb)	24.8	88.1	<24.4 (BDL)
Bismuth (Bi)	<24.1 (BDL)	<24.2 (BDL)	<24.4 (BDL)
Thorium (Th)	2.25	3.95	<0.99 (BDL)
Uranium (U)	1.03	11.2	<0.99 (BDL)

Elements are arranged in ascending atomic number, BDL, below detection limit.

The analysis for PTE concentration in the baseline samples and plant shoot samples was conducted by the RJ Lee Group Laboratory in Pittsburgh, PA, using Inductively Coupled Plasma Atomic Emission Spectroscopy (ICP-AES). According to their report, chemical analysis of plant samples involved three steps: a) sample homogenization – the plant samples were cut, overnight oven dried at 105 °C, and grounded using a mixer mill (Cole-Palmer Model BM-450 with a tungsten carbide jar), b) acid digestion - overnight digestion of homogenized samples with HNO_3_ at 95 °C, filtered through Whatman40 filter paper, then 2 ml of HCl was added, and finally double deionized water was added to bring the sample volume to 50 ml, and c) analysis - done using Inductively coupled plasma atomic emission spectroscopy (ICP-AES) models Agilent 5100, Agilent 5800 and Perkin Elmer Optima 8300.

### 2.5 Data analysis

To investigate the influence of plant species and soil composition on heavy metal accumulation, this study employed a two-way ANOVA design. The dependent variable is the concentration of heavy metals in plant tissues, while the independent variables are plant species and soil treatment. Prior to analysis, the data were assessed for normality, and transformations were applied if necessary to ensure adherence to this assumption. Additionally, the homogeneity of variances across the treatment groups was evaluated. When significant differences were detected in the ANOVA, Tukey’s Honestly Significant Difference (HSD) test was conducted to identify specific pairwise comparisons between plant species and soil treatments. This comprehensive approach allowed for a thorough examination of the interactive effects of these factors on heavy metal accumulation in plants.

## 3 Results

Among the twenty analyzed PTEs, As, Li, and Pb had concentrations below detection limits for all the analyzed plant shoot samples. Similarly, Cr, Mo, Ni, Ti, and V had most of the samples below detection limits across the treatments and species. Hence, further analysis was done for Al, Ba, B, Ca, Cu, Fe, Mg, Mn, S, Si, Sr, and Zn using two-way ANOVA and principal component analysis. **Table 2** shows the detection limit values for each element in plant shoot samples below.

**Table 2:** The detection limits of the non-dominant PTEs in the plant shoot samples.

PTEs	As	Cr	Li	Mo	Ni	Pb	Ti	V
Detection limits	<1.2	<0.5	<0.7	0.5	<0.2	<1.2	<0.5	<0.2

### 3.1 Effect of FA-contamination on the shoot biomass yield

The analysis of variance for the shoot weights of the plants showed a highly significant difference across species, treatments, and their interaction, as shown in **Table 3**. Among the six treatments, 10% FA (T2) had a greater impact on plant biomass accumulation across all species. On the other hand, the control (T1) (i.e., pure soil) yielded higher shoot biomass than the other treatments. However, it did not differ significantly from the FA- and biochar-containing treatments (T4, T5, and T6). Comparison of biochar and FA mixed treatments with the T2 (no biochar) suggests that the addition of biochar to FA-amended soils can increase plant shoot biomass and ameliorate the adverse effects of FA contamination on shoot biomass.

**Table 3:** ANOVA table for shoot biomass.

Source of variation	Sum of squares	df	Mean square	F	P
Species	7543	3	2514.2	69.32	< .001
Treatments	1788	5	357.6	9.86	< .013
Species x treatments	2432	15	162.1	4.47	< .001
Residuals	2611	72	36.3		

The shoot biomass comparison among all species showed lower biomass for hairy vetch and higher biomass for switchgrass. Visual observations in the greenhouse supported these findings (**Supplementary Information**), with hairy vetch and sericea lespedeza exhibiting severe toxicity symptoms and early mortality. This indicates that the grass species, viz., switchgrass and tall fescue, performed better in terms of shoot biomass production and were more tolerant of the adverse experimental conditions in the greenhouse than the legume species, *viz*., hairy vetch and sericea lespedeza.

**Figure 1:**
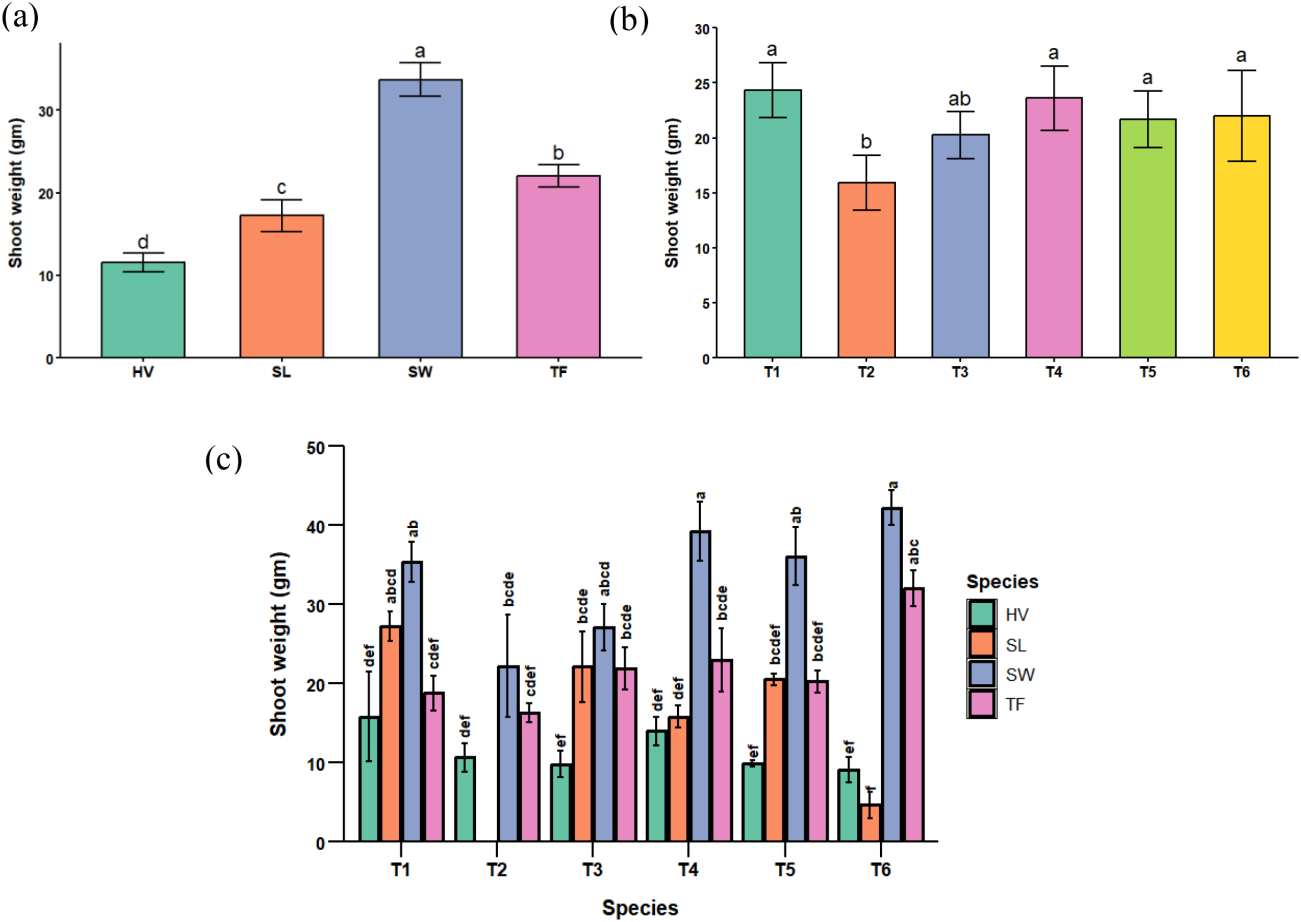
**(a)** effects of species on shoot weights across all treatments, **(b)** effects of treatments on shoot weights across all plant species, and **(c)** effects of treatment x species interaction on shoot weights (SW, Switchgrass; HV, Hairy Vetch; TF, Tall Fescue; and SL, Sericea Lespedeza, T1 = Control, T2 = 10% FA, T3 = 10% BC, T4 = 5% FA + 5% BC, T5 = 2.5% FA + 7.5% BC, and T6 = 7.5% FA + 2.5% BC).

Finally, considering individual species within specific treatments, a higher shoot biomass yield was recorded for switchgrass in the biochar-treated soil mixes. In contrast, a 100% mortality was observed in sericea lespedeza at T2 (10% FA) resulting in lower yield compared to other plant species. The shoot weights of switchgrass, hairy vetch, and tall fescue reduced by 32.82%, 27.01%, and 8.57% in T2 compared to control. However, the increase in biochar proportion in the treatments improved the shoot biomass yields across all plant species indicating an ameliorative role of biochar in the FA-contaminated soils. The results of treatment, species, and interaction effects on the shoot weights are summarized in **Figure 1**.

### 3.2 Effects of treatments on the root biomass yields

Significant differences in root biomass yields were recorded across the species and treatments, as shown in **Table 4**. The root biomass weight results were consistent with the shoot biomass results across the treatments and species. Hairy vetch a had lower root biomass accumulation, followed by sericea lespedeza and tall fescue. On the other hand, switchgrass had a higher biomass weight, demonstrating its better performance and tolerance to PTEs among grass species compared to legume species in FA-contaminated soils.

**Table 4:** ANOVA table for the root biomass yield.

Source of variation	Sum of squares	df	Mean square	F	P
Species	11048	3	3682.7	38.18	< .001
Treatments	1342	5	268.5	2.78	0.024
Species: treatments	1778	15	118.5	1.23	0.271
Residuals	6944	72	96.4		

**Figure 2:**
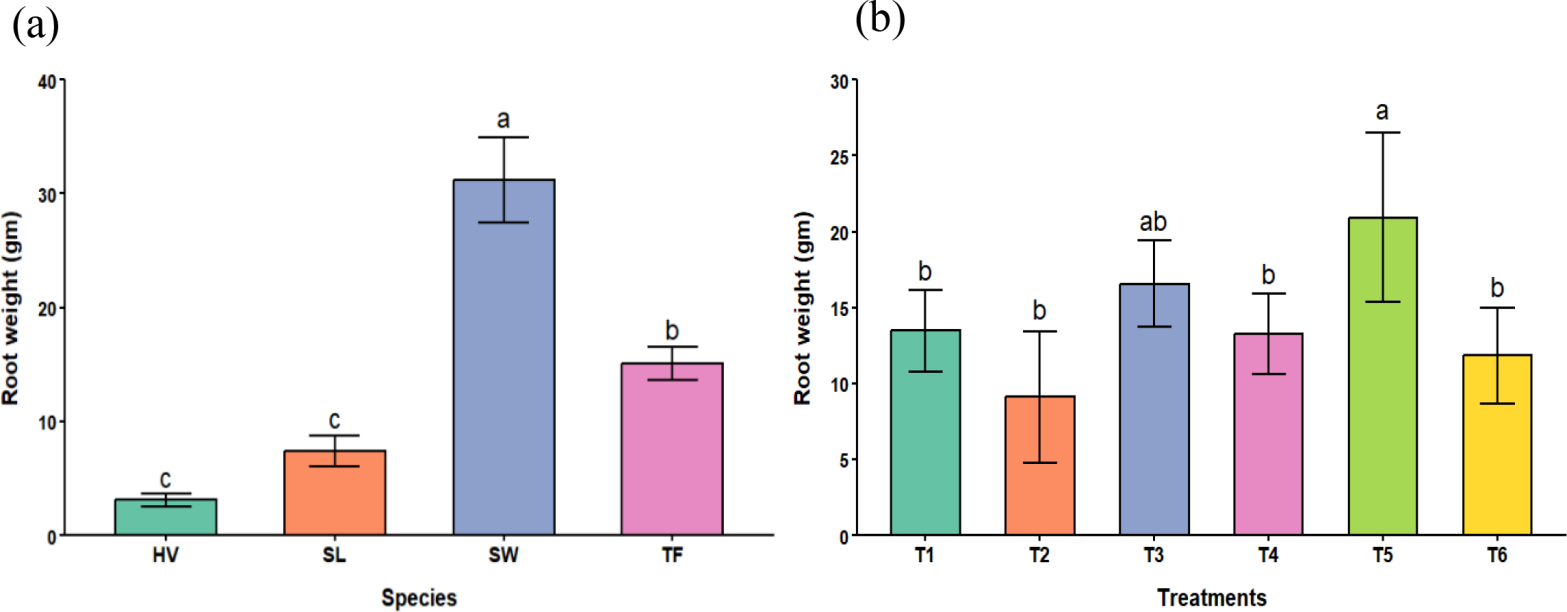
**(a)** effects of species on root weights across all treatments and **(b)** effects of treatments on root weights across all plant species (SW, Switchgrass; HV, Hairy Vetch; TF, Tall Fescue; and SL, Sericea Lespedeza, T1 = Control, T2 = 10% FA, T3 = 10% BC, T4 = 5% FA + 5% BC, T5 = 2.5% FA + 7.5% BC, and T6 = 7.5% FA + 2.5% BC).

A significant difference was also seen across the treatments. Similar to the shoot biomass results, the minimum root biomass weights were recorded in T2, and the maximum weights were seen in T5. Treatments T3 and T5, with 10% and 7.5% biochar, were significantly higher than T2 (no biochar), suggesting that a higher biochar proportion in the treatments provides a suitable environment for root growth. These results are summarized in **Figure 2**.

### 3.3 Effect of treatments on the bioaccumulation of PTEs

The analysis of variance showed significant differences in the bioaccumulation of 6 PTEs (Ba, B, Ca, Cu, Mg, and Sr), as shown in **Table 5**. However, PTEs such as As, Li, and Pb were below detection limits in the plant shoots. A higher bioaccumulation in plants was observed in T2 (10% FA), and a lower concentration in T3 (10% biochar) for most significant PTEs. In addition, the mixed amendments (T4-T6) fall within the concentration ranges of T2 and T3, exhibiting intermediate effects on PTE bioaccumulation in plants. The results indicate that when biochar is amended to the soil with FA, it reduces the bioaccumulation of PTEs in plants. For example, in B, the mean concentration values in plant shoots decrease proportionately with the increase in biochar proportions in soil mixes. An increase in biochar concentration reduces bioaccumulation of PTEs, possibly because it immobilizes PTEs in soil.

**Table 5:** ANOVA table for effects of treatments on bioaccumulation of PTEs.

PTE	Al	Ba	B	Ca	Cu	Fe	Mg	Mn	Si	Sr	S	Zn
P value	0.26	**0.001**	**<0.001**	**0.01**	**<0.001**	0.325	**<0.001**	0.085	0.547	**0.001**	0.13	0.67

Full Individual ANOVA tables are in the **Supplementary Material**.

The bold values represent significance values. It means the potentially toxic elements with those bold values are significantly different across a) treatments.

In the case of Ba and Sr, control showed higher concentration in plant shoots. It is likely due to the higher concentrations of Ba and Sr in the soil used in our study. The treatments T2, T3, T5, and T6 did not exhibit significant variation in Ba and Sr concentrations. However, the Ba concentration in T4 (5% FA + 5% biochar) was significantly lower, suggesting that when equal concentrations of FA and biochar are added to soils, Ba and Sr immobilization increase, thereby reducing their concentrations in plant shoots.

Boron had a different bioaccumulation behavior compared to Ba and Sr. It followed T2 > T6 > T4 > T5 > T1 > T3. It is because the baseline B concentration in FA was very high (544 mg/kg) compared to soil (50.4 mg/kg) and biochar (not detected). Due to a lower baseline concentration, bioaccumulation was lower in treatments with no or lower FA amendment percentages. With decreases in FA concentration and increases in biochar concentration in the treatments, B accumulation in the plant shoots decreases. It can be inferred from this result that, due to the absence or lower concentration of B in biochar, the addition of biochar reduces the baseline concentration of B in FA-contaminated soils and facilitates its immobilization within the soil, ultimately reducing plant-available B.

**Figure 3:**
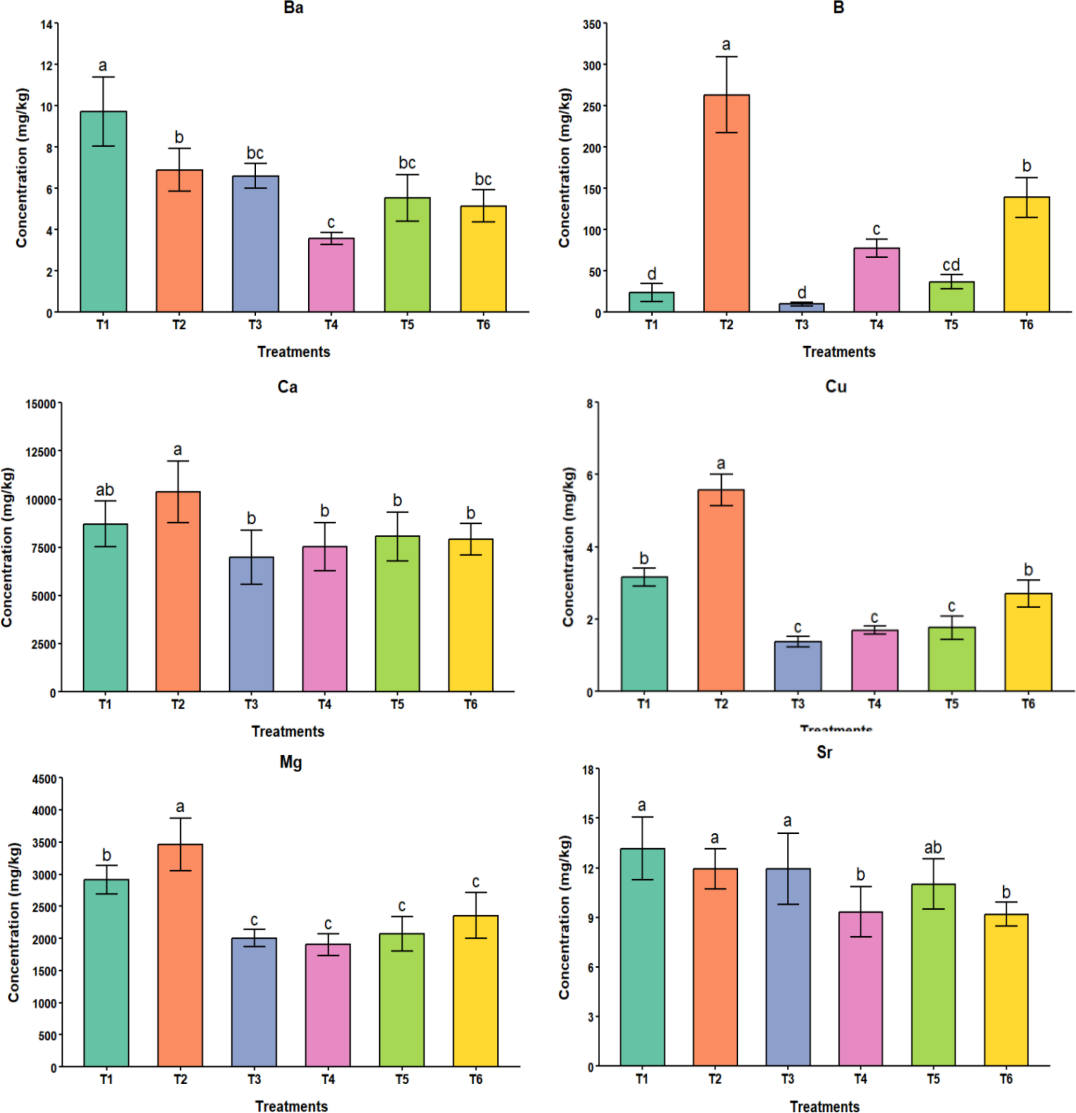
Bar plots showing the effects of treatments on the bioaccumulation of plant species for the significant PTEs (T1 = Control, T2 = 10% FA, T3 = 10% Biochar, T4 = 5% FA + 5% Biochar, T5 = 2.5% FA + 7.5% Biochar, and T6 = 7.5% FA + 2.5% Biochar).

In the case of Ca, a higher bioaccumulation was observed in T2, but it did not differ significantly from the control treatment. There was no significant difference observed across all the biochar amended treatments (T3-T6). It can be inferred from this result that biochar amendment reduces bioaccumulation of Ca in plant shoots; however, the proportion of biochar applied, ranging from 2.5% to 10%, does not significantly differ the bioaccumulation of Ca in plants. There was a significant difference in Cu and Mg concentrations in plant shoots between control and T2, suggesting that a 10% FA addition significantly increased their concentrations. A lower proportion of biochar in T6 (2.5% biochar + 7.5% FA) did not substantially differ from the control in Cu bioaccumulation, indicating that a higher proportion of biochar is needed to stabilize Cu in the soil. The effects of treatments on the bioaccumulation of PTEs are summarized in **Figure 3** graphs.

### 3.4 Effect of species on the bioaccumulation of PTEs

The bioaccumulation of all PTEs differed significantly among species, as shown in **Table 6**. Arsenic, Lithium, and Lead were not detected in the plant shoot samples. Hairy vetch performed better in terms of bio-accumulation for Ca, Sr, S and Zn, whereas tall fescue showed a higher concentration for Al, Ba, B, Cu, Fe, Mg, Mn, and Si. Switchgrass, despite being the best performer in the biomass accumulation in FA treated soils did not accumulate higher concentrations of all the PTEs except for Si in its biomass compared to other plant species in our experiment. This may be due to switchgrass’s extensive root system stabilizing PTEs in the soil (USDA & NRCS, 2011). Another explanation could be the low translocation factor and high bioaccumulation factor of switchgrass roots. A comprehensive analysis of root biomass, shoot biomass, soil mixes, and leachates will be instrumental in determining the mechanisms by which it produces higher yields while accumulating very low concentrations of PTEs in its biomass. It will also help in determining whether this species is a suitable phytostabilizer, phytoextractor, or neither. Sericea lespedeza did not show signs of being a good bioaccumulator of PTEs.

**Table 6:** ANOVA table for effects of species on bioaccumulation of PTEs.

PTE	Al	Ba	B	Ca	Cu	Fe	Mg	Mn	Si	Sr	S	Zn
P value	**0.002**	**0.024**	**0.006**	**<0.001**	**<0.001**	**0.003**	**<0.001**	**0.026**	**0.002**	**<0.001**	**<0.001**	**<0.001**

Full Individual ANOVA tables are in the **Supplementary Material**.

The bold values represent significance values. It means the potentially toxic elements with those bold values are significantly different across b) species.

**Figure 4:**
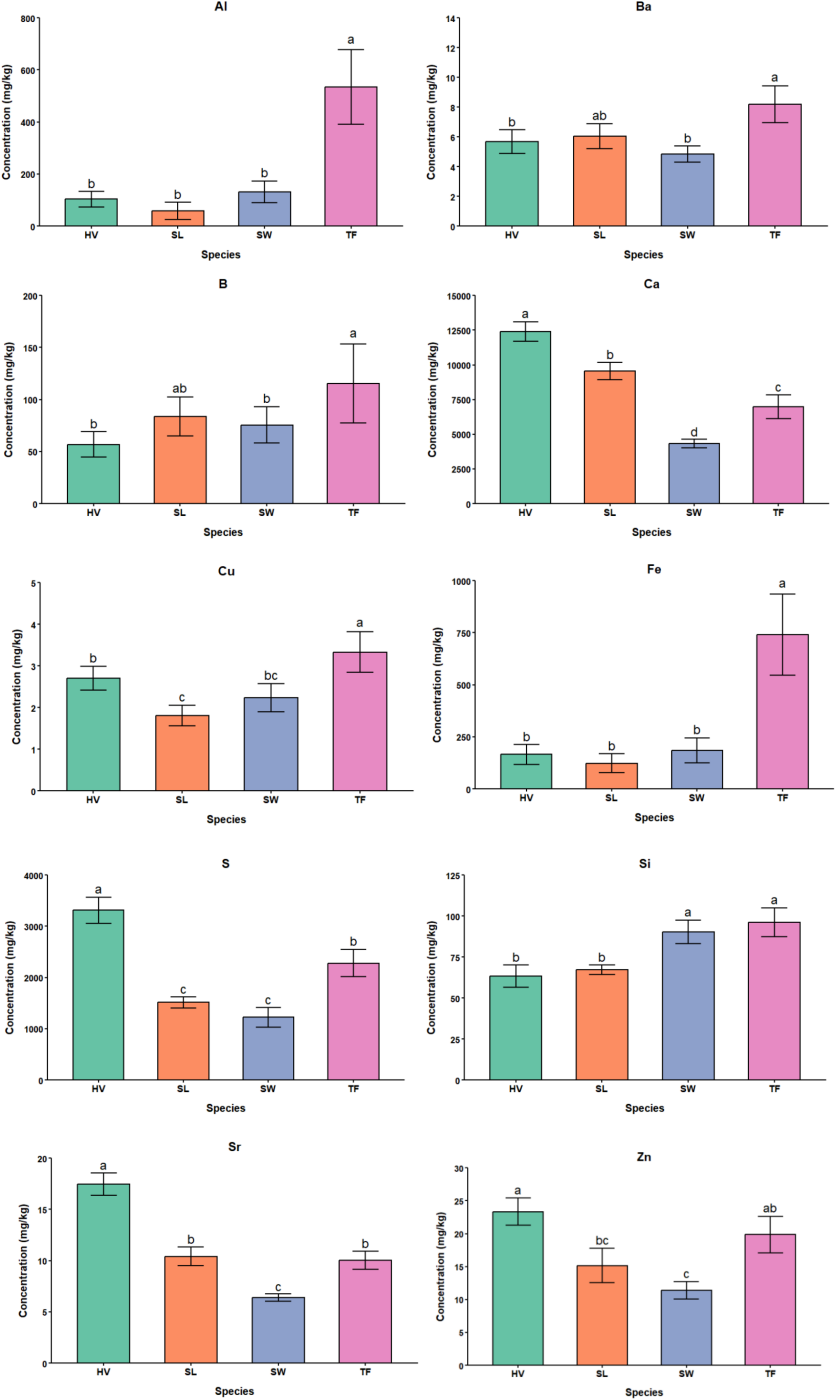
Bar plots showing the effects of species on the bioaccumulation for the significant PTEs in the plant shoot biomass (SW, Switchgrass; HV, HairyVetch; TF, Tall Fescue; and SL, Sericea Lespedeza).

For Si, a clear separation between the grass species and legume species was seen. Both tall fescue and switchgrass accumulated significantly higher concentrations of Si compared to legumes (hairy vetch and sericea lespedeza). This difference can be attributed to the uptake capacity and transport mechanisms of the roots of legumes and grasses (Ma and Takahashi, 2002). Grass species more efficient Si uptake and translocation systems, particularly due to higher transporter capacity and active xylem loading, which results in greater Si bioaccumulation in grasses (Ma and Yamaji, 2006). The effects of species on the bioaccumulation of PTEs are summarized in **Figure 4** graphs.

### 3.5 Effect of species x treatment interactions on the bioaccumulation of PTEs

**Figure 5:**
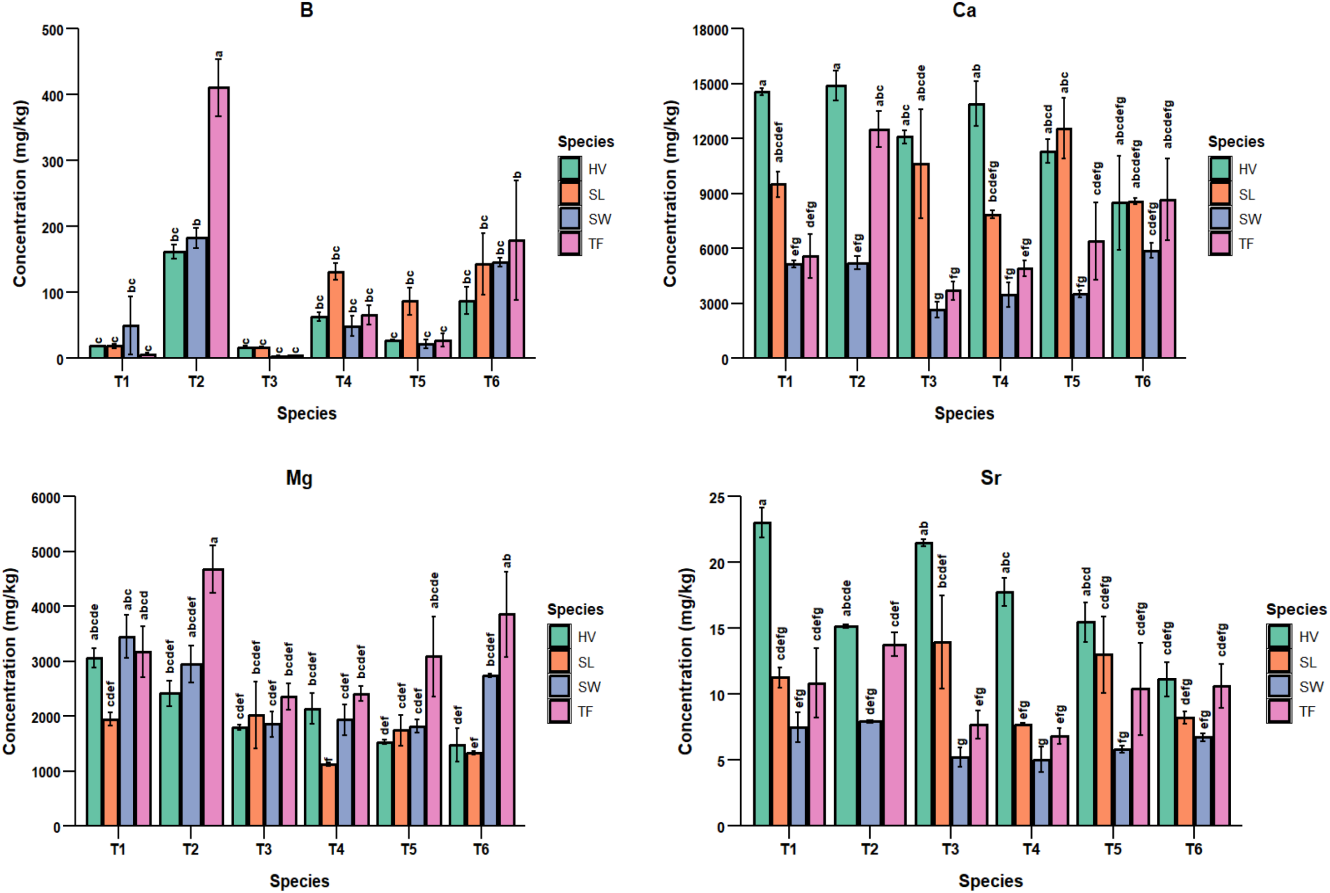
Bar plots showing the effects of interaction between species and treatments on the bioaccumulation of PTEs by plant species B, Ca, Mg, and Sr (SW, Switchgrass; HV, Hairy Vetch; TF, Tall Fescue; and SL, Sericea Lespedeza; T1 = Control, T2 = 10% FA, T3 = 10% Biochar, T4 = 5% FA + 5% Biochar, T5 = 2.5% FA + 7.5% Biochar, and T6 = 7.5% FA + 2.5% Biochar).

The interaction between treatments and plant species was significant for the bioaccumulation of B, Ca, Mg, and Sr, as shown in **Table 7**. A higher bioaccumulation was observed in tall fescue in T2 for B and Mg, hairy vetch in T2 for Ca, and hairy vetch in the control for Sr. On the other hand, switchgrass showed lower concentrations of B, Ca, and Sr at 10% BC, and sericea lespedeza showed the minimum at T4 (5% FA + 5% BC). These results indicate that the best-performing species x treatment interactions for PTE bioaccumulation are tall fescue and hairy vetch at 10% FA. In contrast, switchgrass and sericea lespedeza at 10% BC showed poor shoot PTE bioaccumulation. The interactions indicate that higher biochar concentrations reduce PTE bioaccumulation in plants, potentially creating a more favorable environment for their growth and development. Hence, biochar can be used as a soil amendment in various phytoremediation through phytostabilization. However, biochar is not recommended for phytoextraction projects. The effects of treatment species interactions are summarized in **Figure 5** graphs.

**Table 7:** ANOVA table for Effect of interaction between species and treatments on the bioaccumulation of PTEs.

PTE	Al	Ba	B	Ca	Cu	Fe	Mg	Mn	Si	Sr	S	Zn
P value	0.981	0.507	**<0.001**	**<0.001**	0.311	0.972	**0.015**	0.891	0.614	**<0.001**	0.245	0.114

Full Individual ANOVA tables are in the **Supplementary Material**.

The bold values represent significance values. It means the potentially toxic elements with those bold values are significantly different across c) treatment x species interaction.

### 3.6 Principal component analysis

Principal component analysis (PCA) was applied to reduce dimensionality and to explore multivariate relationships among PTE variables derived from their accumulation in the plant species. For paired elements, balances were calculated as proportions of the form A/(A+B), generating four two-element balances: Al–Si, Ca–Mg, Ba–Sr, and Fe–S. Each balance represents the relative dominance of one element over its geochemically associated counterpart, allowing interpretation in terms of mineralogical dominance (Al–Si), alkaline earth behavior (Ca–Mg), trace element substitution, more specifically, isovalent substitution between Ba and Sr (Ba–Sr), and redox-sensitive processes, such as bacterial reactions (Fe–S). To integrate the remaining micronutrients, a grouped balance was calculated as (Cu+Zn)/(Cu+Zn+Mo+B), representing the relative dominance of Cu and Zn versus Mo and B within the combined micronutrient pool. This balance captures shifts in micronutrient partitioning rather than absolute concentrations. PCA was conducted using the correlation matrix, and the first two principal components were retained for interpretation and visualization, as they explained the majority of variance in the dataset. PCA biplots were used to simultaneously examine sample clustering by species and the contribution of individual balance variables to observed multivariate patterns.

**Figure 6:**
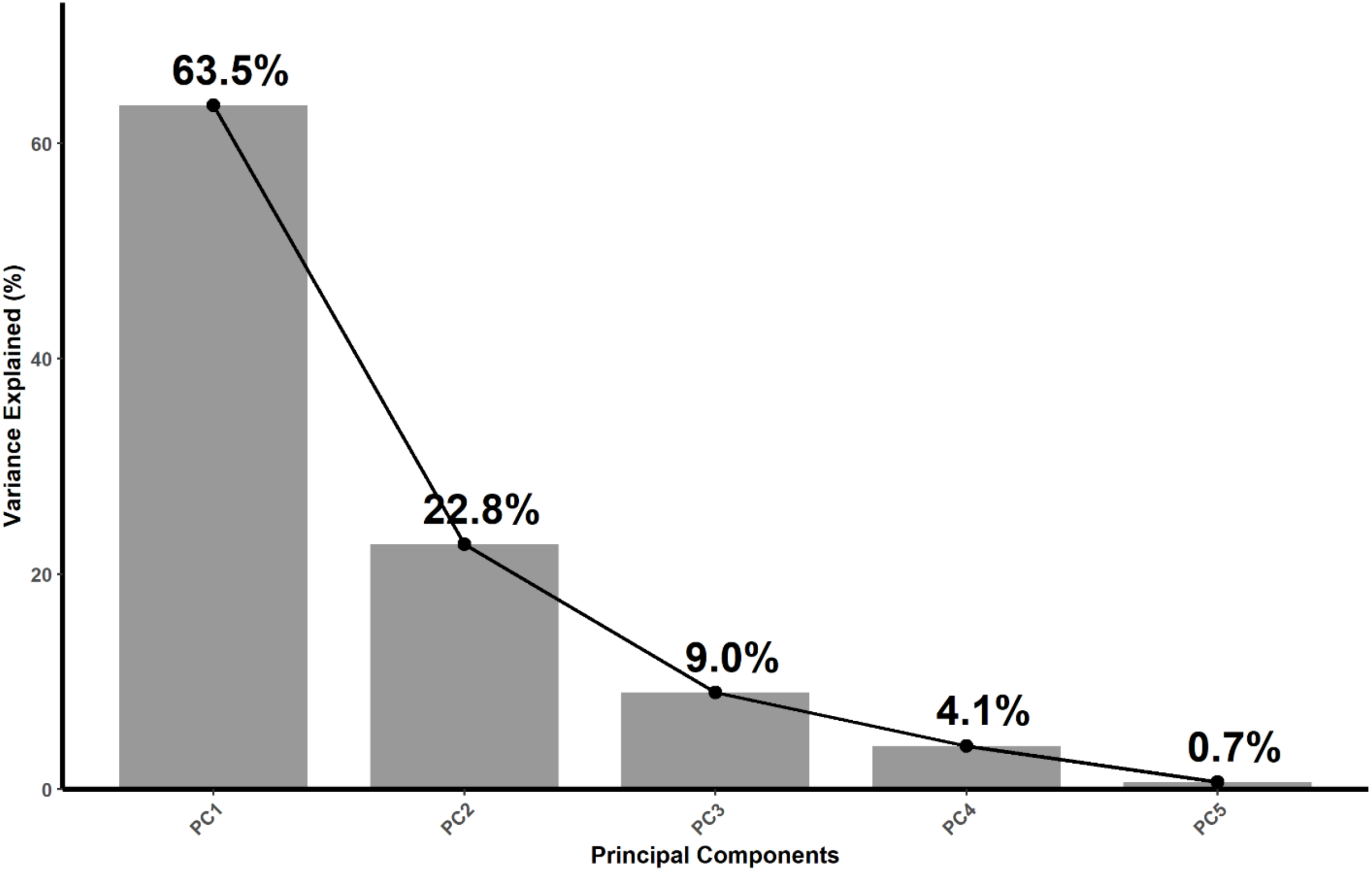
Scree plot of principal components. The sharp drop in variance after PC1 (64%) and PC2 (23%) indicates that these two components capture the major structure of the dataset, with later components contributing only marginal variance.

**Figure 7:**
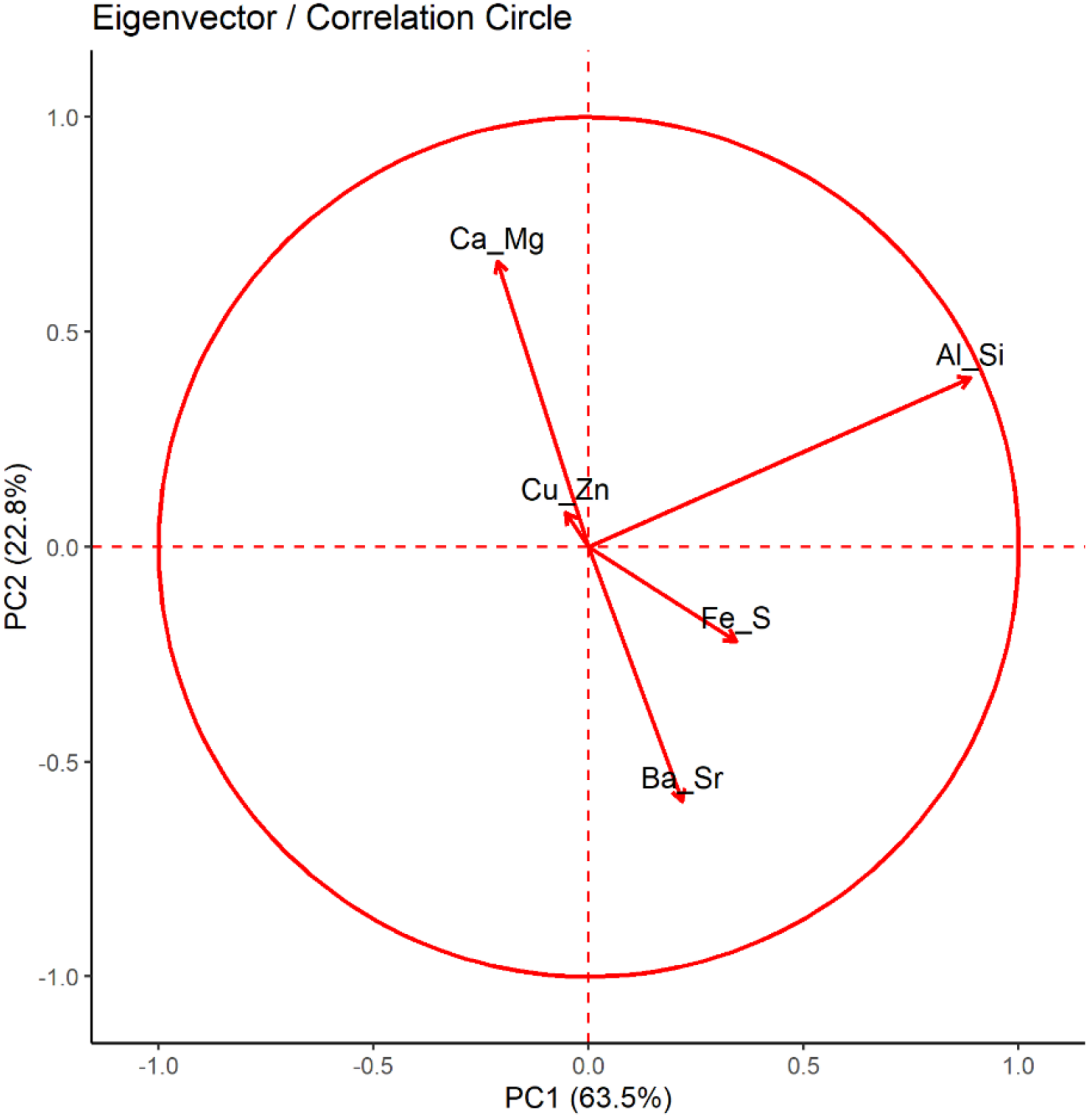
PCA correlation circle showing relationship among elemental groupings (SW, Switchgrass; HV, Hairy Vetch; TF, Tall Fescue; and SL, Sericea Lespedeza, T1 = Control, T2 = 10% FA, T3 = 10% Biochar, T4 = 5% FA + 5% Biochar, T5 = 2.5% FA + 7.5% Biochar, and T6 = 7.5% FA + 2.5% Biochar).

**Figure 8:**
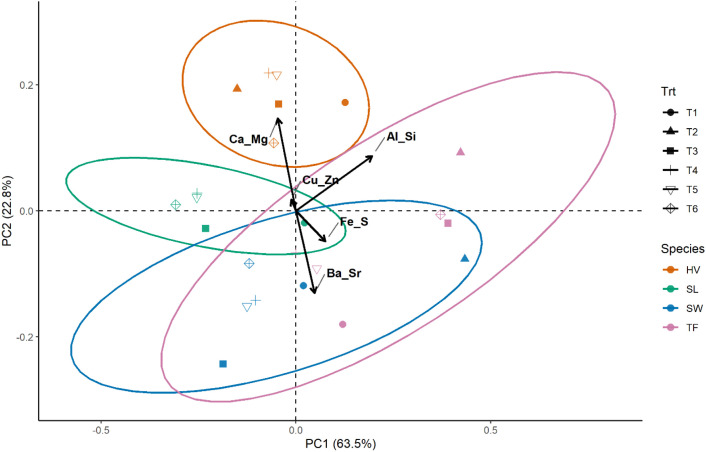
PCA biplot showing the distribution of plant species (HV, SL, SW, TF) relative to elemental bioaccumulation patterns (SW, Switchgrass; HV, Hairy Vetch; TF, Tall Fescue; and SL, Sericea Lespedeza, T1 = Control, T2 = 10% FA, T3 = 10% Biochar, T4 = 5% FA + 5% Biochar, T5 = 2.5% FA + 7.5% Biochar, and T6 = 7.5% FA + 2.5% Biochar, ellipses represent the 95% confidence regions for each species).

**Figure 9:**
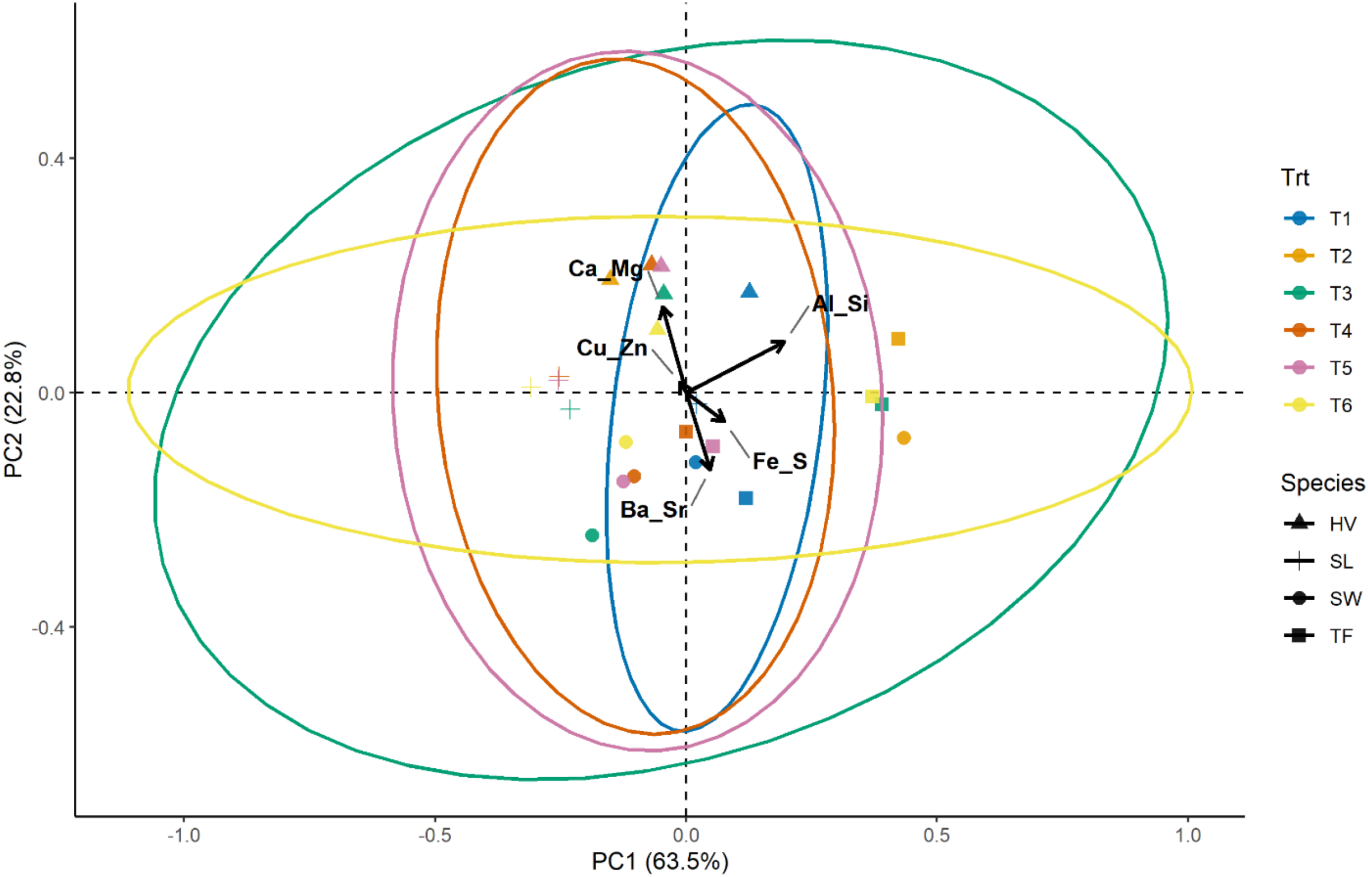
PCA biplot illustrating the effect of soil treatments on elemental bioavailability and plant uptake patterns (SW, Switchgrass; HV, Hairy Vetch; TF, Tall Fescue; and SL, Sericea Lespedeza, T1 = Control, T2 = 10% FA, T3 = 10% Biochar, T4 = 5% FA + 5% Biochar, T5 = 2.5% FA + 7.5% Biochar, and T6 = 7.5% FA + 2.5% Biochar, ellipses represent the 95% confidence regions for each species).

PCA grouped the variables into components. The first two components (PC1 and PC2) explained 87% of the total variance, and the subsequent components accounted for the remaining 13%, as shown in **Figure 6**. The principal component 1 (PC1) was dominated by Al-Si association with secondary contributions from Fe-S, whereas PC2 was dominated by Ca-Mg association in the positive direction and Ba-Sr balance in the negative direction (**Figure 7**). The Cu-Zn-Mo-B association had minimal contribution to both PC1 and PC2. The results show that Ca-Mg and Ba-Sr association are strongly and negatively related with each other. They belong to the same group in the periodic table but reflect contrasting cation regulation strategies. Calcium and Mg represent actively regulated base nutrients, whereas Ba and Sr behave as chemically analogous but biologically non-essential cations; moreover, they represent carbonate and phosphate associations, respectively, in the soil. Their relationship suggests that the bioaccumulation of Ca and Mg is associated with reduced Ba and Sr uptake in plants and reflect selective cation discrimination. On the other hand, The orthogonal relationship between the Ca-Mg – Ba-Sr axis and Al-Si axis shows that these elements are largely uncorrelated in their bioaccumulation patterns in plants. It can be inferred from this result that the biological cation regulation (Ca-Mg) and mineral phase (Al-Si) operate as independent processes. The Fe-S association demonstrated a moderate positive correlation with Ba-Sr. The PCA biplots (**Figures 8**, **9**) revealed that the elemental bioaccumulation was primarily driven rather than treatment driven, highlighting the dominant role of intrinsic species traits in governing multivariate elemental uptake behavior. There was a clear separation of the grass and legume species for their elemental uptake behavior in their biomasses. Legumes (hairy vetch and sericea lespedeza) showed convergence towards Ca-Mg association, whereas grasses showed their clustering towards Ba-Sr association, indicating underlining contrasting cation regulation strategies. Hairy vetch showed a strong association with Ca-Mg and clear expulsion of Ba-Sr, while tall fescue showed a broad association, but weak Ca-Mg alignment. Switchgrass was primarily linked to Fe-S and Ba-Sr bioaccumulation, and sericea lespedeza showed a moderate association of Fe-S and Cu-Zn-Mn-B groups. The larger ellipse sizes for grass species demonstrate greater variability in their elemental uptake patterns, whereas legumes showed selective and more constrained uptake strategies.

## 4 Discussion

Tall fescue and hairy vetch have shown promising results in this experiment in terms of bioaccumulation of potentially toxic elements in the fly ash-treated soils. Tall fescue bioaccumulates higher concentrations of Al, Ba, B, Cu, Fe, Mg, Mn, and Si compared to other species studied in our research. Conversely, Hairy vetch accumulates Ca, Sr, S, and Zn in higher concentrations. These species have demonstrated their ability to accumulate a wide range of PTEs, indicating their potential for use in phytoremediation of sites contaminated with various PTEs. However, their biomass yield decreased in the FA-amended soils compared to the control soils, while PTE accumulation in their biomass increased. This suggests that they are not highly tolerant of PTE contamination with FA. Further studies on their tolerance abilities or the levels of FA tolerance are needed to determine whether they can be used on a large scale for site remediation of heavily contaminated sites.

Sericea lespedeza is typically found growing in disturbed areas where other types of vegetation are minimal (Texas A&M Agrilife Extension, n.d.). Nevertheless, it did not perform well in these experiments, with 100% mortality observed in the 10% FA-amended soil mix. A temporal repetition of this experiment is needed to validate this unexpected result. A hypothesis for this unexpected result is that the presence of a wide range of PTEs in FA limits its nutrient uptake capacity. There were 36 PTEs analyzed in the soil + FA + biochar combinations, and among them, some might be very toxic, lethally toxic, causing all the plants to die. Another explanation can be the physical properties of FA that limit its growth and development. The 10% FA treatment caused the soil to be very hard, making it difficult for the plant roots to penetrate. Also, soil pH, which increases with FA addition in the soil, was not considered. Sericea lespedeza prefers a slightly acidic environment for its proper growth and development (Vandevender, 2002). The higher pH caused by FA might have been lethal to its growth in our experiment.

In this study, FA demonstrated highly negative impacts on plant germination, growth, and development. A 100% mortality of sericea lespedeza in 10% FA-amended soil in our experiment corroborated this statement. However, our results indicate that the addition of biochar reduces the negative impacts of PTE contamination with FA. Novak et al. (2019) noted that adding biochar to a mine site contaminated with Cd and Zn increased the switchgrass growth and yield. Biochar not only increases biomass yield but also stabilizes PTEs in the soil, reducing their mobility in the soil and absorption by the plants. According to Ibrahim et al., 2022, biochar reduces Pb, Cd, Zn, and Cu concentrations in the plant shoots by 89.2%, 37.2%, 35.5%, and 40.2%, respectively. It changes the oxidation states of PTEs, reducing their toxicity and increasing their mobility (Shi et al., 2022). For example, it reduces the oxidation state of Cr from Cr^6+^ to Cr^3+^ through continuous transfer of electrons, possibly due to functional groups on biochar surfaces that contain oxygen, where the latter oxidation state of Cr is less mobile and toxic (Narayanan and Ma, 2022; Shi et al., 2022; Xu et al., 2019).

When multiple types of PTEs are present in the soil, the bioavailability of each element changes (Kumpiene et al., 2008; Lee et al., 2023). For example, in this study, it was found that the bioavailability of Ca-Mg had strong negative correlation with Ba-Sr. Menzel, 1954 studied the competitive relationship between Ca and Sr accumulation in plants and stated that the ratio of Ca and Sr in plants is equal to a constant multiplied by the ratio of Ca and Sr in the soil, underlining the negative interaction of Ca and Sr uptake in plants. In contrast, Cooke and Leishman, 2016 reported the bioaccumulation of Ca and Si are not correlated further corroborating the results of our study. The uptake of Si and S also showed a weaker relationship in this study, supported by the findings of Teixeira et al., 2020. The PTE bioaccumulation also greatly differs across the plant species. For instance, Si uptake depends upon the density of Si-transporter in plant roots that transport Si from soil solution to the cortical cells and to the xylem, and this density is found higher in grass species of Gramineae and Cyperaceae families (Ma and Yamaji, 2006). Another study also reported that Si accumulation is higher in monocot plants such as grasses to improve structural support tolerance ability of plants to diseases and drought (Hodson et al., 2005). It supports our findings of higher Si accumulation in tall fescue and switchgrass than hairy vetch and sericea lespedeza. On the other hand, White and Broadley, 2003 categorizes legumes as calcicoles/calcitrophs – species that occur on calcareous soils – and state that these species have a higher soluble Ca concentration. They accumulate higher Ca concentrations in shoots compared to grasses and their differences are linked to differences in cell wall properties, particularly the higher pectin in dicots such as legumes content. Pectin provides negatively charged binding sites for Ca retention through cation exchange in cell walls, and hence the lower pectin content in grasses lead to reduced Ca accumulation than legumes (White and Broadley, 2003).

## 5 Limitations and future research directions

There are a few limitations to this research. First, the analysis for PTEs included only the plants’ aboveground parts. Budgetary constraints limited the analysis of roots, soil mixes, and leachates for twenty PTEs at a commercial laboratory, resulting in a limited data set. Comprehensive data is needed to calculate various indices, such as the bioaccumulation factor (BAF) and the translocation factor (TF), which determine the efficiency of a phytoremediation project. These indices are also crucial in determining whether a plant is a phytostabilizer or a phytoextractor. Similarly, vegetative and reproductive parts of the plants were not separated into the samples. Analyzing the vegetative and reproductive parts of plants would help identify the bioaccumulation characteristics of plant species. It is beneficial in post-phytoextraction processes, such as phytomining. If it is well known where plants concentrate most of their PTEs, it will be valuable to facilitate the economic extraction of elements such as lithium, nickel, gold, and rare earth elements from these plants. In addition, this research does not discuss the fate of contaminated plant biomasses. The scope of this research is limited to the bioaccumulation of PTEs by plants, but it does not cover how those plants should be managed or disposed of safely.

## 6 Conclusion

This study showed a significant difference between grass and legume species in FA–amended soils with biochar application. Grass species, namely switchgrass and tall fescue, consistently produced higher biomass than legumes (hairy vetch and sericea lespedeza) across all treatments, that indicates their greater tolerance to the PTE contamination by FA. Fly ash contamination considerably reduced the plant biomasses for all the species; however, biochar amendment in FA-contaminated soil enhanced the plant root and shoot growth. The PCA showed a distinct separation of bioaccumulation behavior of PTEs across the species but there was no clear separation across the treatments. Calcium and Mg showed a strong negative correlation with Ba-Sr and a weak correlation with Al-Si. Grasses (switchgrass and tall fescue) showed a higher bioaccumulation of Si, whereas grasses, particularly hairy vetch, showed a higher concentration of Ca in its shoot biomass. Across the treatments, biochar amended treatments showed a reduced concentrations of PTEs in the plant shoots, potentially through their stabilization in the soil. Due to its role in reducing PTE bioaccumulation in plant shoots, biochar is not an effective soil amendment for phytoextraction.

## Data Availability

The original contributions presented in the study are included in the article/**Supplementary Material**. Further inquiries can be directed to the corresponding author.
