# Supplementary material for: Effects of biochar amendment on the phytoextraction of twenty potentially toxic elements in fly ash contaminated soils
Source: Front Plant Sci. 2026 Mar 31;17:1783879. doi: 10.3389/fpls.2026.1783879 (PMC13076276; doi:10.3389/fpls.2026.1783879)
Supplement: Supplementary file 1 [file DataSheet1.pdf]

Supplementary Materials (ANOVA)

ANOVA tables for Plant bioaccumulation of PTEs

1. Aluminum (Al)

ANOVA table:

Source	Df	Sum Sq	Mean Sq	F value	Pr(>F)
Trt	5	920354	184071	1.355	0.26023
Species	3	2285801	761934	5.607	**0.00246**
Trt:Species	14	669404	47815	0.352	0.98133
Residuals	43	5842817	135879		

Post hoc for Species (DMRT):

	Mean_Al	groups
TF	534.05	a
SW	131.4778	b
HV	103.2647	b
SL	58.50769	b

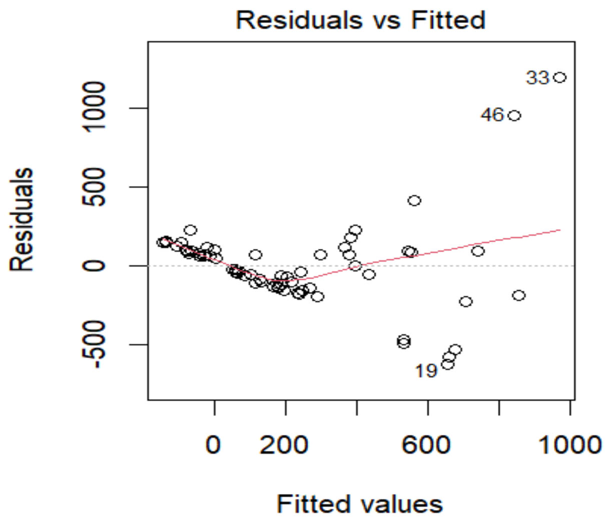

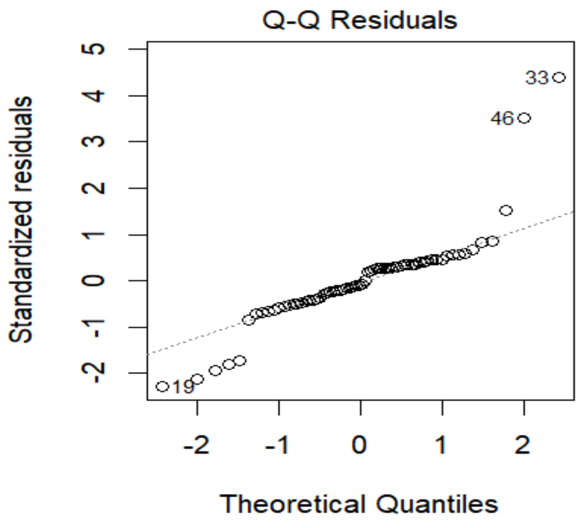

2. Barium (Ba)
ANOVA table:

**Source**	**Df**	**Sum Sq**	**Mean Sq**	**F value**	**Pr(>F)**
Trt	5	253.3	50.66	4.841	0.00134 **
Species	3	108.8	36.28	3.466	0.02421 *
Trt:Species	14	140.6	10.04	0.959	0.50780
Residuals	43	450.0	10.46		

Post hoc analysis for Treatments:

**Trt**	**Mean Ba**	**Group**
Control	9.7000	a
10FA	6.8875	b
10BC	6.6000	bc
2.5FA+7.5BC	5.5364	bc
7.5FA+2.5BC	5.1500	bc
5FA+5BC	3.5750	c

Post hoc analysis for Species:

**Species**	**Mean Ba**	**Group**
TF	8.1944	a
SL	6.0538	ab
HV	5.6647	b
SW	4.8444	b

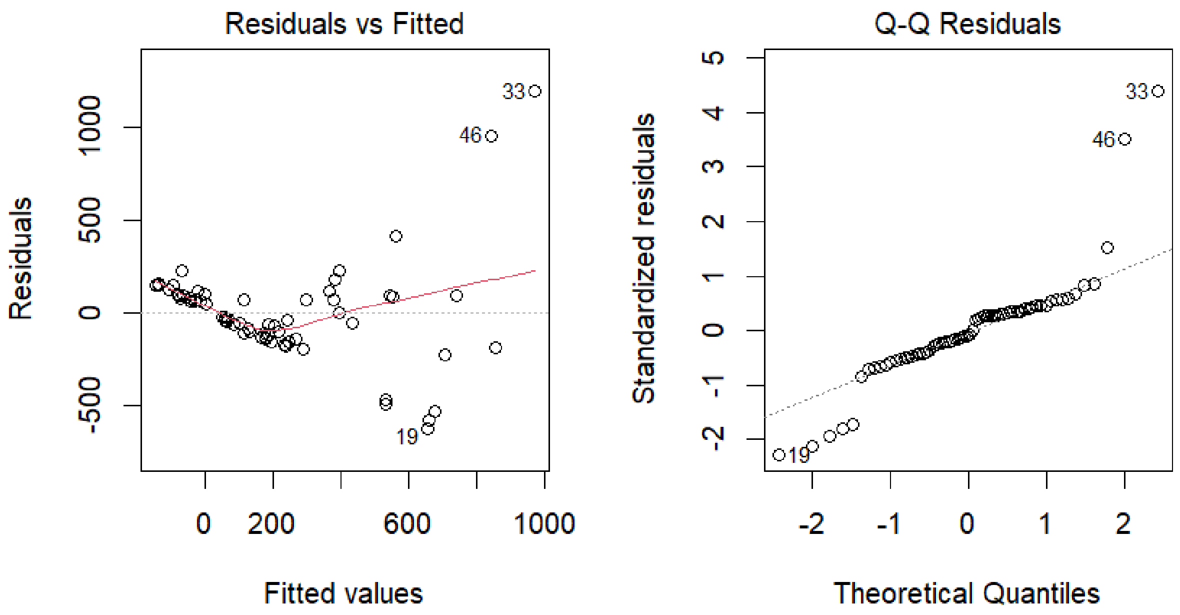

3. Boron (B)

Source	Df	Sum Sq	Mean Sq	F value	Pr(>F)
Trt	5	421828	84366	37.962	1.03e-14 ***
Species	3	31314	10438	4.697	0.006349 **
Trt:Species	14	108838	7774	3.498	0.000779 ***
Residuals	43	95561	2222		

Post hoc analysis for Trt:

Treatment	B	groups
10FA	263.0000	a
7.5FA+2.5BC	138.9417	b
5FA+5BC	77.4833	c
2.5FA+7.5BC	36.8000	cd
Control	23.6417	d
10BC	9.7909	d

Post hoc analysis for Species:

Species	B	groups
TF	115.5778	a
SL	83.8308	a
SW	75.5278	a
HV	56.9177	a

Post hoc analysis for the interaction:

Trt	Species	ppm	groups
10FA	TF	410.667	a
10FA	SW	183.000	b
7.5FA+2.5BC	TF	178.867	b
10FA	HV	161.500	bc
7.5FA+2.5BC	SW	145.667	bc
7.5FA+2.5BC	SL	143.533	bc
5FA+5BC	SL	131.667	bcd
7.5FA+2.5BC	HV	87.700	cde
2.5FA+7.5BC	SL	86.750	cdef
5FA+5BC	TF	65.833	def
5FA+5BC	HV	63.433	def
Control	SW	50.400	ef
5FA+5BC	SW	49.000	ef
2.5FA+7.5BC	HV	27.800	ef
2.5FA+7.5BC	TF	27.700	ef
2.5FA+7.5BC	SW	21.600	ef
Control	HV	19.133	ef
Control	SL	18.733	ef
10BC	SL	17.250	ef
10BC	HV	16.800	ef
Control	TF	6.300	f
10BC	TF	4.100	f
10BC	SW	3.500	f

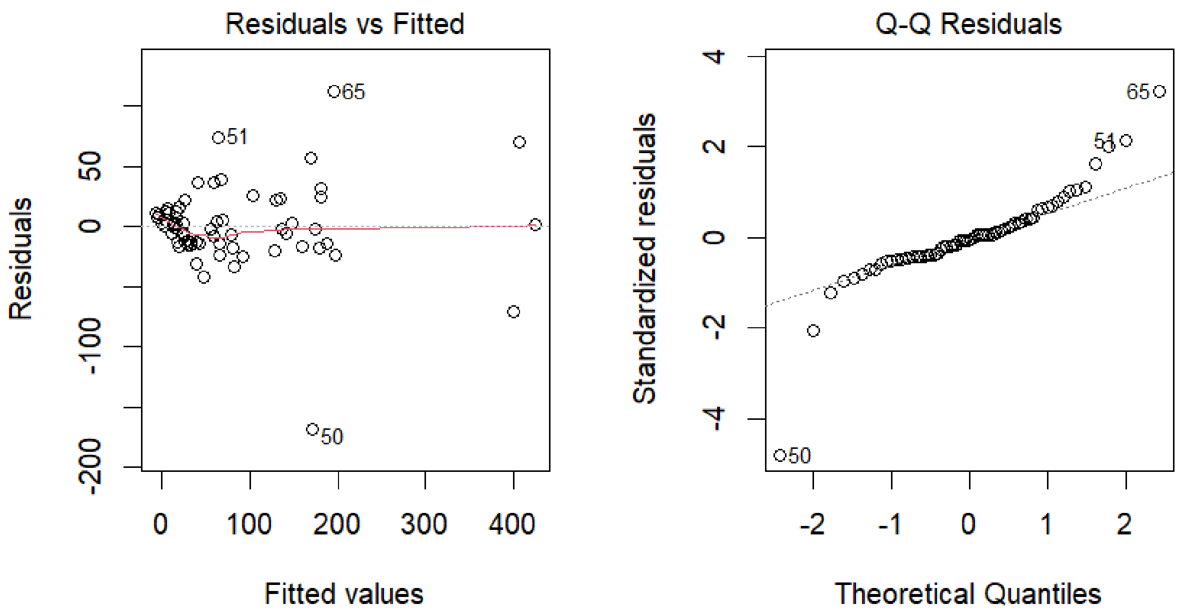

4. Calcium (Ca)
ANOVA

Source	Df	Sum Sq	Mean Sq	F value	Pr(>F)
Trt	5	63,803,089	12,760,618	3.446	0.010471 *
Species	3	663,469,474	221,156,491	59.724	2.23×10^{-15} ***
Trt:Species	14	186,039,552	13,288,539	3.589	0.000619 ***
Residuals	43	159,226,733	3,702,947		

Post hoc for treatments:

Treatment	ppm	groups
10FA	10370.000	a
Control	8709.167	ab
2.5FA + 7.5BC	8067.273	b
7.5FA + 2.5BC	7909.167	b
5FA + 5BC	7536.667	b
10BC	6969.091	b

Post hoc for species:

Species	ppm	groups
HV	12405.294	a
SL	9553.077	b
TF	6966.111	c
SW	4319.444	d

Post hoc for interaction:

Trt : Species	ppm	groups
10FA : HV	14900.000	a
Control : HV	14566.667	a
5FA+5BC : HV	13900.000	ab
2.5FA+7.5BC : SL	12550.000	abc
10FA : TF	12500.000	abc
10BC : HV	12100.000	abc
2.5FA+7.5BC : HV	11300.000	bcd
10BC : SL	10620.000	bcde
Control : SL	9506.667	cdef
7.5FA+2.5BC : TF	8680.000	defg
7.5FA+2.5BC : SL	8580.000	defg
7.5FA+2.5BC : HV	8496.667	defg
5FA+5BC : SL	7863.333	efgh
2.5FA+7.5BC : TF	6400.000	fghi
7.5FA+2.5BC : SW	5880.000	ghi
Control : TF	5603.333	ghij

Trt : Species	ppm	groups
10FA : SW	5220.000	hij
Control : SW	5160.000	hij
5FA+5BC : TF	4910.000	hij
10BC : TF	3703.333	ij
2.5FA+7.5BC : SW	3513.333	ij
5FA+5BC : SW	3473.333	ij
10BC : SW	2670.000	j

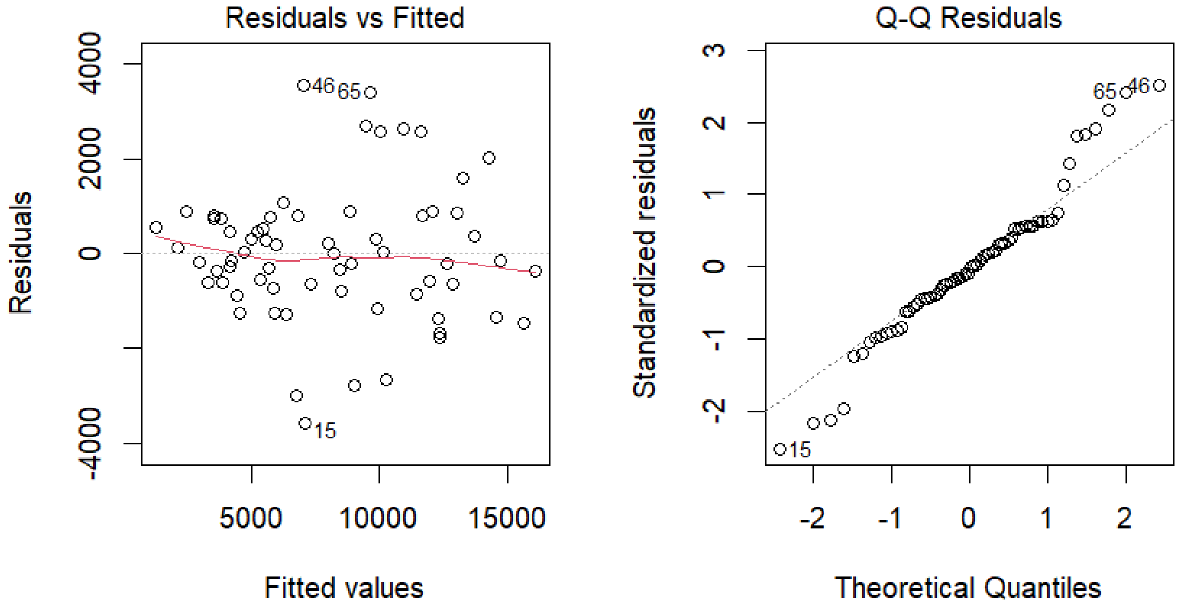

5. Copper (Cu)
ANOVA table:

Source	Df	Sum Sq	Mean Sq	F value	Pr(>F)
Trt	5	108.77	21.755	33.300	9.63×10^{-14} ***
Species	3	13.91	4.636	7.096	5.61×10^{-4} ***
Trt:Species	14	10.96	0.783	1.198	0.311

Source	Df	Sum Sq	Mean Sq	F value	Pr(>F)
Residuals	43	28.09	0.653		

Post hoc: Treatments

Treatment	ppm	Group
10FA	5.57500	a
Control	3.15833	b
7.5FA+2.5BC	2.70000	b
2.5FA+7.5BC	1.76364	c
5FA+5BC	1.69167	c
10BC	1.37273	c

Post hoc: Species

Species	ppm	Group
TF	3.33333	a
HV	2.70588	ab
SW	2.23333	b
SL	1.80769	b

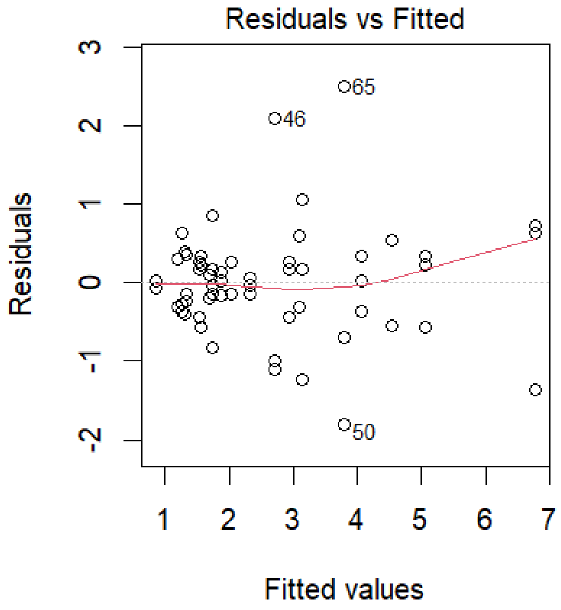

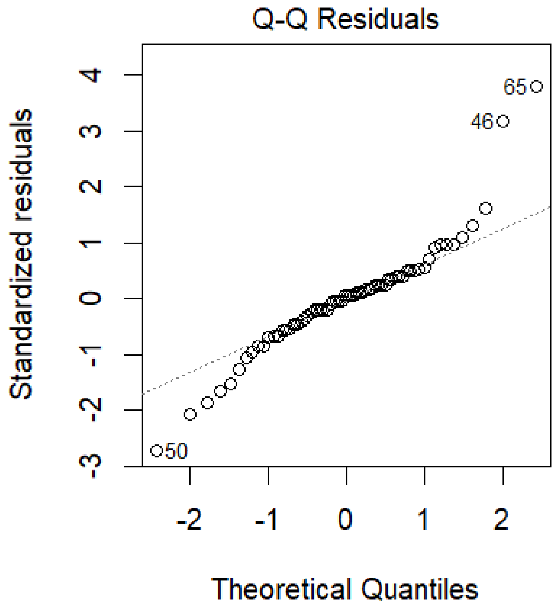

6. Iron (Fe)
ANOVA table:

	Df	Sum Sq	Mean Sq	F value	Pr(>F)
Trt	5	1548496	309699	1.201	0.32509
Species	3	4108260	1369420	5.309	0.00334**
Trt:Species	14	1391817	99416	0.385	0.97205
Residuals	43	11092105	257956		

Post hoc: Species

Species	ppm	groups
TF	740.5556	a
SW	185.6222	b
HV	165.7059	b
SL	123.2308	b

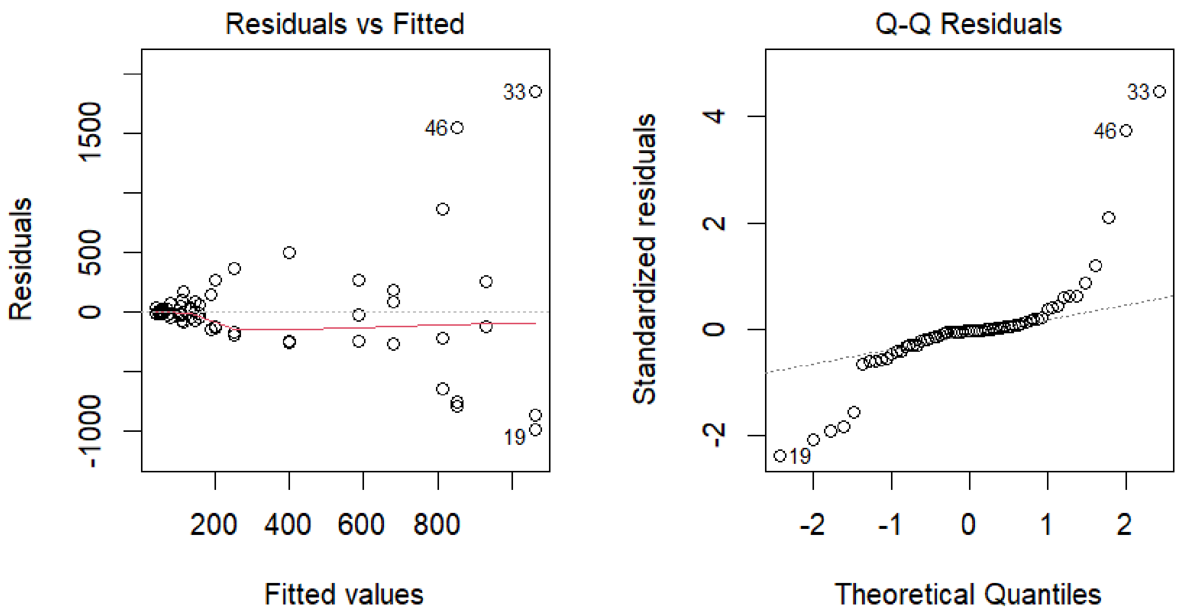

7. Magnesium (Mg)
ANOVA table:

**Source**	**Df**	**Sum Sq**	**Mean Sq**	**F value**	**Pr(>F)**
Trt	5	18039812	3607962	10.628	1.09e-06 ***
Species	3	20350048	6783349	19.982	2.91e-08 ***
Trt:Species	14	11294392	806742	2.376	0.015 *
Residuals	43	14597650	339480		

Post hoc: Treatments

**Treatment**	**ppm**	**groups**
10FA	3462.500	a
Control	2910.000	ab
7.5FA+2.5BC	2352.500	bc
2.5FA+7.5BC	2072.727	c
10BC	2005.455	c
5FA+5BC	1902.500	c

Post hoc: Species

Species	ppm	groups
TF	3260.000	a
SW	2457.778	b
HV	2050.588	bc
SL	1596.923	c

Post hoc: Interaction

Trt	Species	ppm	groups
10FA	TF	4676.667	a
7.5FA+2.5BC	TF	3853.333	ab
Control	SW	3453.333	bc
Control	TF	3176.667	bcd
2.5FA+7.5BC	TF	3086.667	bcde
Control	HV	3063.333	bcde
10FA	SW	2943.333	bcde
7.5FA+2.5BC	SW	2740.000	cdef
10FA	HV	2420.000	cdefg
5FA+5BC	TF	2413.333	defg
10BC	TF	2353.333	defg
5FA+5BC	HV	2136.667	efgh
10BC	SL	2020.000	efghi
Control	SL	1946.667	fghi
5FA+5BC	SW	1936.667	fghi
10BC	SW	1856.667	fghi
2.5FA+7.5BC	SW	1816.667	fghi
10BC	HV	1796.667	fghi

Trt	Species	ppm	groups
2.5FA+7.5BC	SL	1745.000	fghi
2.5FA+7.5BC	HV	1533.333	ghi
7.5FA+2.5BC	HV	1476.667	ghi
7.5FA+2.5BC	SL	1340.000	hi
5FA+5BC	SL	1123.333	i

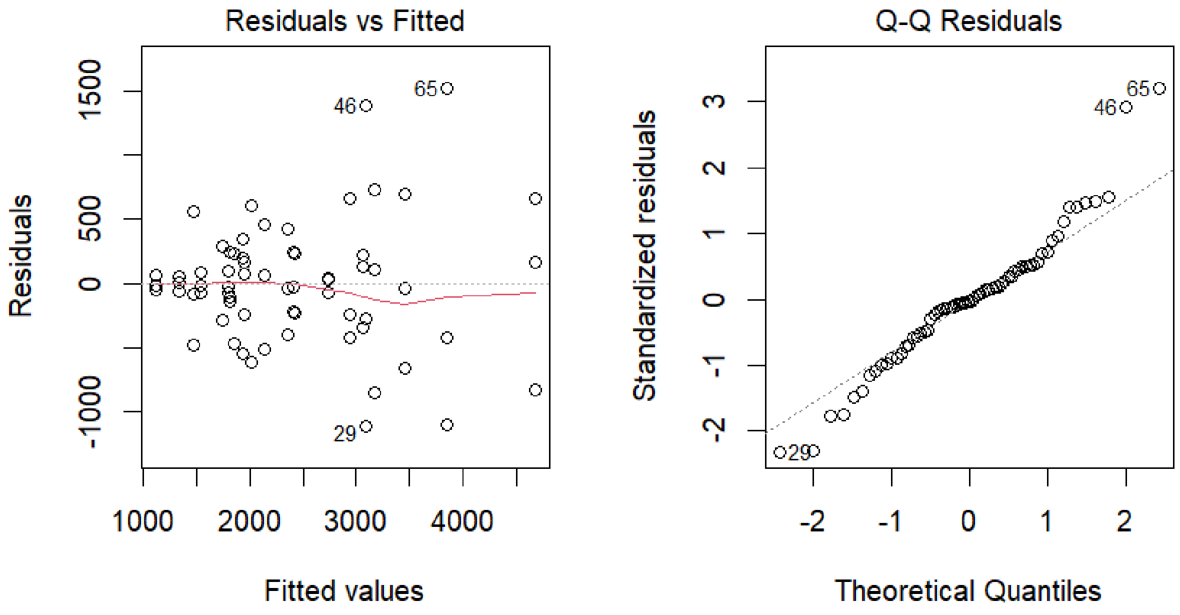

8. Manganese (Mn)
ANOVA table:

Source	Df	Sum Sq	Mean Sq	F value	Pr(>F)
Trt	5	17801	3560	2.090	0.0851 .
Species	3	17325	5775	3.390	0.0264 *
Trt:Species	14	13015	930	0.546	0.8907
Residuals	43	73255	1704		

Post hoc: Species

Species	ppm	Group
TF	72.5000	a
SL	51.3615	ab
HV	38.7176	b
SW	31.4167	b

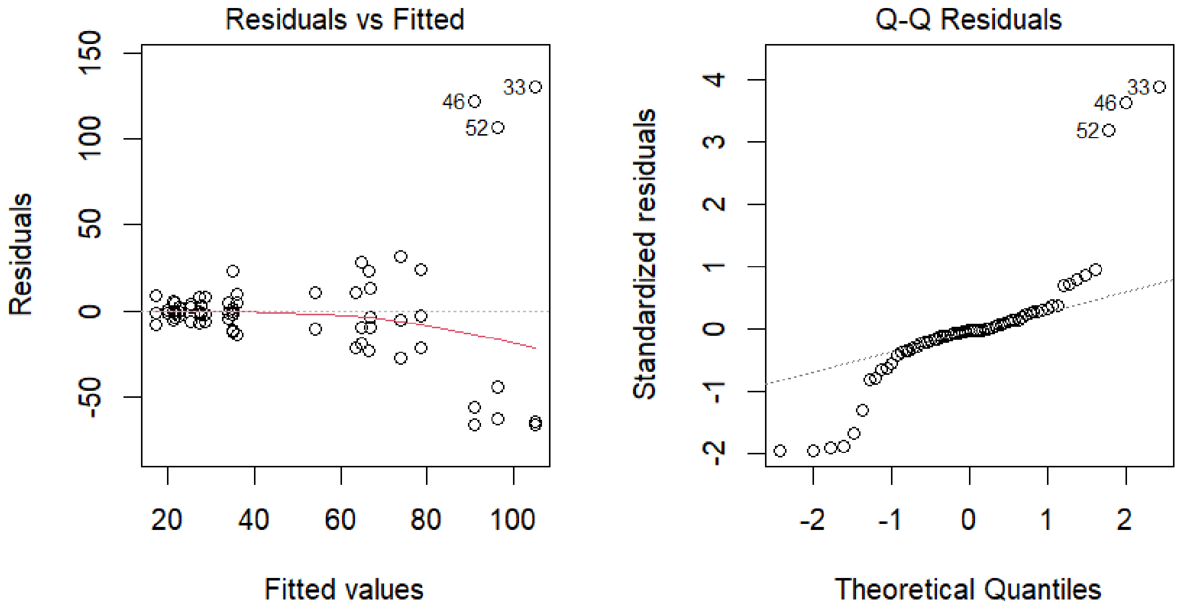

9. Silicon (Si)
ANOVA table:

	Df	Sum Sq	Mean Sq	F value	Pr(>F)
Trt	5	3 581	716	0.813	0.54681
Species	3	14 563	4 854	5.512	0.00271 **
Trt:Species	14	10 481	749	0.850	0.61413
Residuals	43	37 867	881		

Post hoc: Species

Species	ppm	groups
TF	96.14444	a
SW	90.34444	a
SL	67.36154	b
HV	63.34118	b

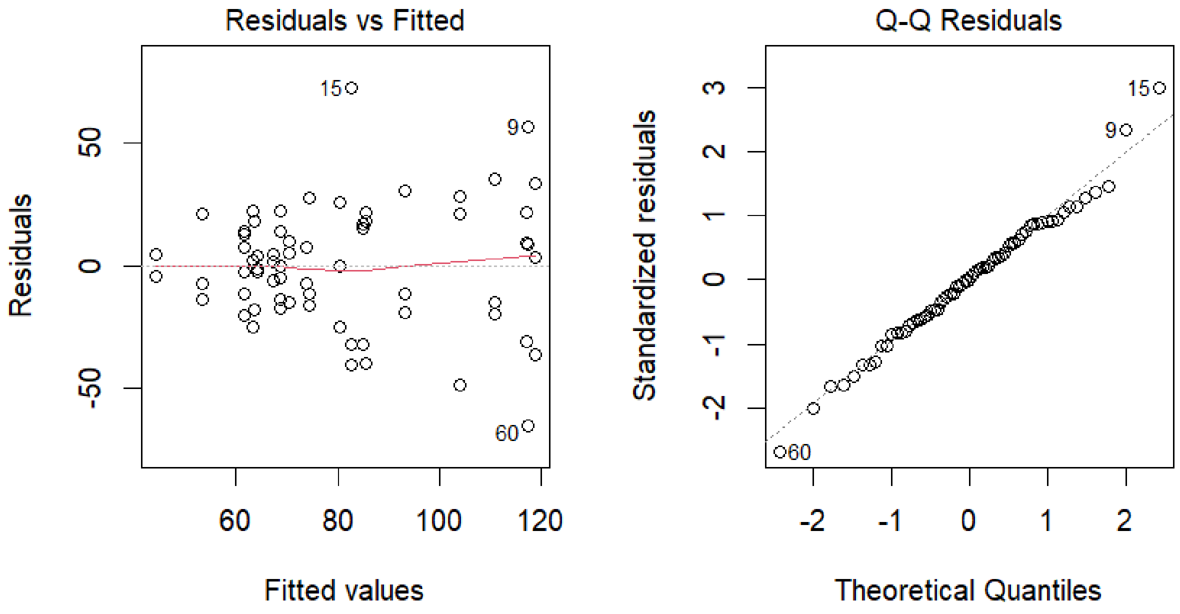

10. Strontium (Sr)
ANOVA table:

Source	Df	Sum Sq	Mean Sq	F value	Pr(>F)
Trt	5	145.4	29.1	5.010	0.001054 **
Species	3	1126.7	375.6	64.695	5.54e-16 ***
Trt:Species	14	320.1	22.9	3.938	0.000259 ***
Residuals	43	249.6	5.8		

Post hoc: Treatments

Treatment	ppm	Group
Control	13.158333	a
10BC	11.927273	a
10FA	11.925000	a
2.5FA+7.5BC	11.009091	a
5FA+5BC	9.325000	a
7.5FA+2.5BC	9.183333	a

Post hoc: Species

Species	ppm	Group
HV	17.464706	a
SL	10.423077	b
TF	10.027778	b
SW	6.377778	c

Post hoc: Interaction

Treatment:Species	ppm	Group
Control:HV	23.033333	a
10BC:HV	21.500000	ab
5FA+5BC:HV	17.733333	bc
2.5FA+7.5BC:HV	15.466667	cd
10FA:HV	15.150000	cde
10BC:SL	13.950000	cdef
10FA:TF	13.766667	def
2.5FA+7.5BC:SL	13.000000	def
Control:SL	11.266667	efg
7.5FA+2.5BC:HV	11.133333	efg
Control:TF	10.833333	efg
7.5FA+2.5BC:TF	10.633333	fgh

Treatment:Species	ppm	Group
2.5FA+7.5BC:TF	10.400000	fgh
7.5FA+2.5BC:SL	8.233333	ghi
10FA:SW	7.933333	ghi
10BC:TF	7.700000	ghi
5FA+5BC:SL	7.700000	ghi
Control:SW	7.500000	ghi
5FA+5BC:TF	6.833333	hi
7.5FA+2.5BC:SW	6.733333	hi
2.5FA+7.5BC:SW	5.833333	i
10BC:SW	5.233333	i
5FA+5BC:SW	5.033333	i

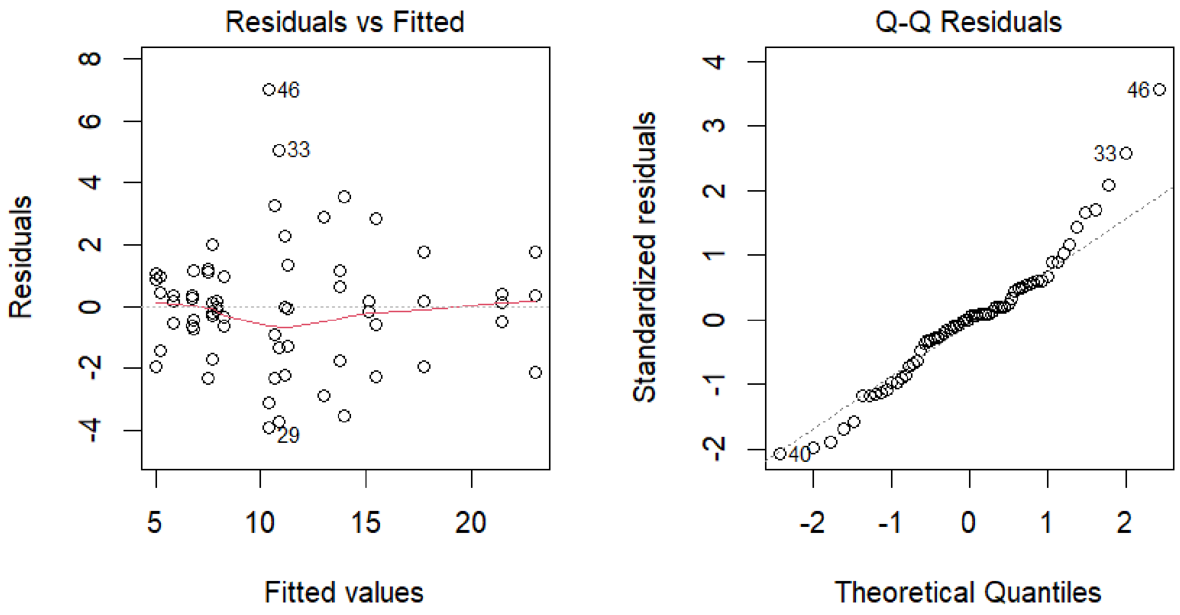

11. Sulfur (S)

ANOVA table

Factor	Df	Sum Sq	Mean Sq	F value	Pr(>F)
Trt	5	6,863,843	1,372,769	1.817	0.130
Species	3	43,786,186	14,595,395	19.321	4.39×10^{-8} ***
Trt:Species	14	13,791,053	985,075	1.304	0.245
Residuals	43	32,482,711	755,412		

Post hoc: Species

Species	ppm	Group
HV	3313.706	a
TF	2282.222	b
SL	1515.000	c
SW	1223.444	c

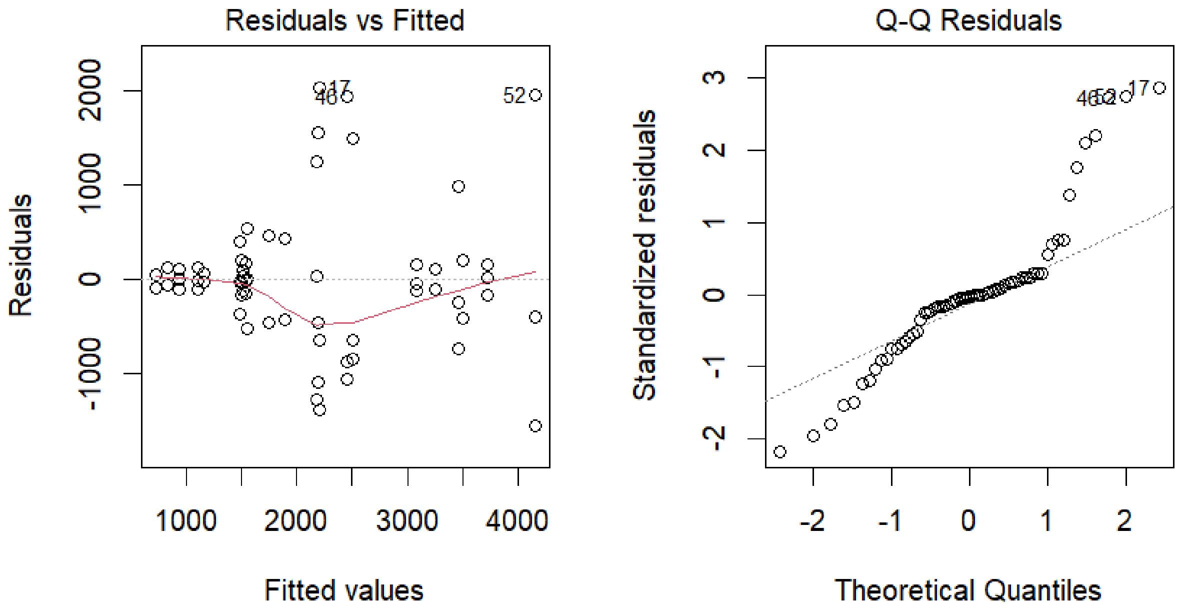

12. Zinc (Zn)
ANOVA table:

Source	Df	Sum Sq	Mean Sq	F value	Pr(>F)
Trt	5	234.3	46.9	0.640	0.670089
Species	3	1428.9	476.3	6.509	0.000995***
Trt:Species	14	1653.7	118.1	1.614	0.114134
Residuals	43	3146.8	73.2		

Post hoc: Species

Species	ppm	groups
HV	23.35294	a
TF	19.86111	ab
SL	15.14615	bc
SW	11.41667	c

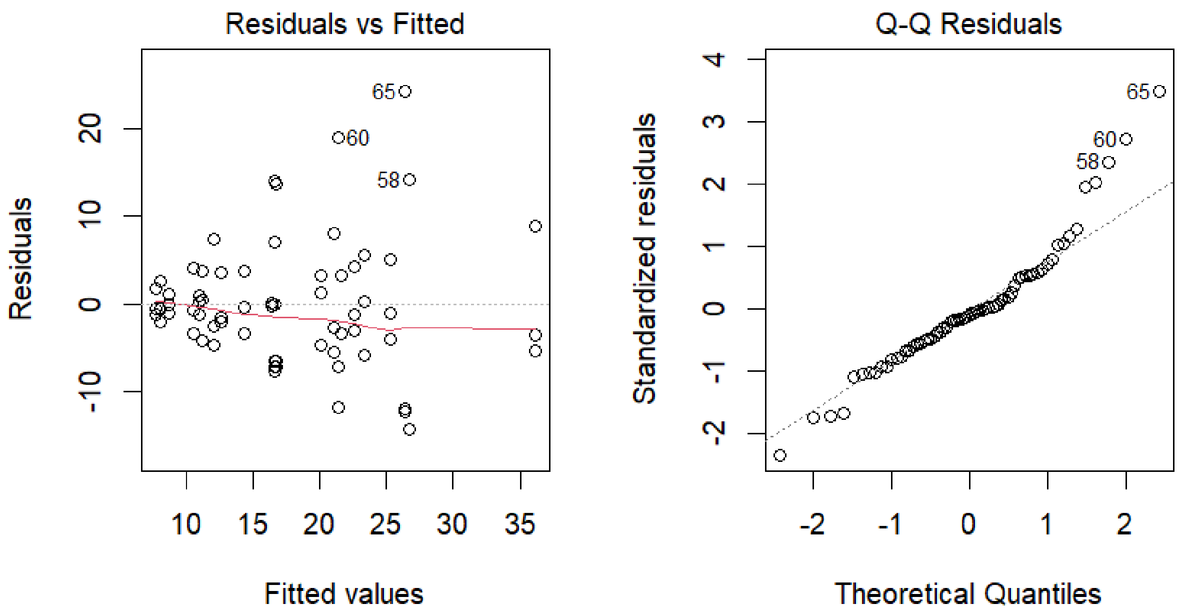

ANOVA tables of Biomass weights

1. Plant Shoots

ANOVA - Shoot_wt

	Sum of Squares	df	Mean Square	F	p
Species	7543	3	2514.2	69.32	< .001
Treatments	1788	5	357.6	9.86	< .001
Species	2432	15	162.1	4.47	< .001
Residuals	2611	72	36.3		

Post Hoc Tests

Post Hoc Comparisons - Species

Comparison							
**Species**		**Species**	**Mean Difference**	**SE**	**df**	**t**	p_{tukey}
SW	-	HV	23.62	1.74	72.0	13.58	< .001
	-	TF	11.67	1.74	72.0	6.71	< .001
	-	SL	18.64	1.74	72.0	10.72	< .001
HV	-	TF	-11.94	1.74	72.0	-6.87	< .001
	-	SL	-4.98	1.74	72.0	-2.86	0.027
TF	-	SL	6.96	1.74	72.0	4.00	< .001

Note. Comparisons are based on estimated marginal means

Post Hoc Comparisons - Soil Mix

Comparison							
**Soil Mix**		**Soil Mix**	**Mean Difference**	**SE**	**df**	**t**	p_{tukey}
TR1	-	TR2	13.366	2.13	72.0	6.2773	< .001
	-	TR3	4.073	2.13	72.0	1.9130	0.403
	-	TR4	2.191	2.13	72.0	1.0289	0.907
	-	TR5	2.622	2.13	72.0	1.2314	0.820
	-	TR6	2.297	2.13	72.0	1.0788	0.888
TR2	-	TR3	-9.293	2.13	72.0	-4.3643	< .001

Post Hoc Comparisons - Soil Mix

Comparison							
Soil Mix		Soil Mix	Mean Difference	SE	df	t	p_{tukey}
	-	TR4	-11.175	2.13	72.0	-5.2485	< .001
	-	TR5	-10.744	2.13	72.0	-5.0459	< .001
	-	TR6	-11.069	2.13	72.0	-5.1986	< .001
TR3	-	TR4	-1.883	2.13	72.0	-0.8841	0.949
	-	TR5	-1.451	2.13	72.0	-0.6816	0.983
	-	TR6	-1.776	2.13	72.0	-0.8342	0.960
TR4	-	TR5	0.431	2.13	72.0	0.2025	1.000
	-	TR6	0.106	2.13	72.0	0.0499	1.000
TR5	-	TR6	-0.325	2.13	72.0	-0.1526	1.000

Note. Comparisons are based on estimated marginal means

Post Hoc Comparisons - Species ✻ Soil Mix

Comparison									
Species	Soil Mix		Species	Soil Mix	Mean Difference	SE	df	t	p_{tukey}
SW	TR1	-	SW	TR2	13.1875	4.26	72.0	3.09683	0.267
		-	SW	TR3	8.2750	4.26	72.0	1.94323	0.951
		-	SW	TR4	-3.8375	4.26	72.0	-0.90116	1.000
		-	SW	TR5	-0.7125	4.26	72.0	-0.16732	1.000
		-	SW	TR6	-6.8375	4.26	72.0	-1.60566	0.994
		-	HV	TR1	19.5750	4.26	72.0	4.59682	0.004
		-	HV	TR2	30.0500	4.26	72.0	7.05667	< .001
		-	HV	TR3	25.5425	4.26	72.0	5.99817	< .001
		-	HV	TR4	24.8625	4.26	72.0	5.83849	< .001
		-	HV	TR5	25.4875	4.26	72.0	5.98526	< .001
		-	HV	TR6	26.2500	4.26	72.0	6.16431	< .001
		-	TF	TR1	16.5625	4.26	72.0	3.88939	0.037
		-	TF	TR2	19.1125	4.26	72.0	4.48821	0.005
		-	TF	TR3	13.4875	4.26	72.0	3.16728	0.231

Post Hoc Comparisons - Species ✻ Soil Mix

Comparison									
Species	Soil Mix		Species	Soil Mix	Mean Difference	SE	df	t	p_{tukey}
		-	TF	TR4	12.4625	4.26	72.0	2.92658	0.368
		-	TF	TR5	15.1375	4.26	72.0	3.55475	0.093
		-	TF	TR6	3.3500	4.26	72.0	0.78668	1.000
		-	SL	TR1	8.1375	4.26	72.0	1.91094	0.958
		-	SL	TR2	35.3875	4.26	72.0	8.31008	< .001
		-	SL	TR3	13.2625	4.26	72.0	3.11445	0.258
		-	SL	TR4	19.5500	4.26	72.0	4.59095	0.004
		-	SL	TR5	14.8500	4.26	72.0	3.48724	0.110
		-	SL	TR6	30.7000	4.26	72.0	7.20931	< .001
	TR2	-	SW	TR3	-4.9125	4.26	72.0	-1.15361	1.000
		-	SW	TR4	-17.0250	4.26	72.0	-3.99800	0.027
		-	SW	TR5	-13.9000	4.26	72.0	-3.26415	0.187
		-	SW	TR6	-20.0250	4.26	72.0	-4.70249	0.003
		-	HV	TR1	6.3875	4.26	72.0	1.49998	0.998
		-	HV	TR2	16.8625	4.26	72.0	3.95984	0.030
		-	HV	TR3	12.3550	4.26	72.0	2.90134	0.385
		-	HV	TR4	11.6750	4.26	72.0	2.74165	0.496
		-	HV	TR5	12.3000	4.26	72.0	2.88842	0.393
		-	HV	TR6	13.0625	4.26	72.0	3.06748	0.283
		-	TF	TR1	3.3750	4.26	72.0	0.79255	1.000
		-	TF	TR2	5.9250	4.26	72.0	1.39137	0.999
		-	TF	TR3	0.3000	4.26	72.0	0.07045	1.000
		-	TF	TR4	-0.7250	4.26	72.0	-0.17025	1.000
		-	TF	TR5	1.9500	4.26	72.0	0.45792	1.000
		-	TF	TR6	-9.8375	4.26	72.0	-2.31015	0.797
		-	SL	TR1	-5.0500	4.26	72.0	-1.18590	1.000
		-	SL	TR2	22.2000	4.26	72.0	5.21325	< .001
		-	SL	TR3	0.0750	4.26	72.0	0.01761	1.000
		-	SL	TR4	6.3625	4.26	72.0	1.49411	0.998
		-	SL	TR5	1.6625	4.26	72.0	0.39041	1.000

Post Hoc Comparisons - Species ✻ Soil Mix

Comparison									
**Species**	**Soil Mix**		**Species**	**Soil Mix**	**Mean Difference**	**SE**	**df**	**t**	**p_{tukey}**
		-	SL	TR6	17.5125	4.26	72.0	4.11248	0.019
	TR3	-	SW	TR4	-12.1125	4.26	72.0	-2.84439	0.423
		-	SW	TR5	-8.9875	4.26	72.0	-2.11054	0.897
		-	SW	TR6	-15.1125	4.26	72.0	-3.54888	0.094
		-	HV	TR1	11.3000	4.26	72.0	2.65359	0.560
		-	HV	TR2	21.7750	4.26	72.0	5.11345	< .001
		-	HV	TR3	17.2675	4.26	72.0	4.05494	0.022
		-	HV	TR4	16.5875	4.26	72.0	3.89526	0.036
		-	HV	TR5	17.2125	4.26	72.0	4.04203	0.023
		-	HV	TR6	17.9750	4.26	72.0	4.22109	0.013
		-	TF	TR1	8.2875	4.26	72.0	1.94616	0.950
		-	TF	TR2	10.8375	4.26	72.0	2.54498	0.640
		-	TF	TR3	5.2125	4.26	72.0	1.22406	1.000
		-	TF	TR4	4.1875	4.26	72.0	0.98335	1.000
		-	TF	TR5	6.8625	4.26	72.0	1.61153	0.994
		-	TF	TR6	-4.9250	4.26	72.0	-1.15654	1.000
		-	SL	TR1	-0.1375	4.26	72.0	-0.03229	1.000
		-	SL	TR2	27.1125	4.26	72.0	6.36686	< .001
		-	SL	TR3	4.9875	4.26	72.0	1.17122	1.000
		-	SL	TR4	11.2750	4.26	72.0	2.64772	0.565
		-	SL	TR5	6.5750	4.26	72.0	1.54401	0.997
		-	SL	TR6	22.4250	4.26	72.0	5.26609	< .001
	TR4	-	SW	TR5	3.1250	4.26	72.0	0.73385	1.000
		-	SW	TR6	-3.0000	4.26	72.0	-0.70449	1.000
		-	HV	TR1	23.4125	4.26	72.0	5.49798	< .001
		-	HV	TR2	33.8875	4.26	72.0	7.95784	< .001
		-	HV	TR3	29.3800	4.26	72.0	6.89934	< .001
		-	HV	TR4	28.7000	4.26	72.0	6.73965	< .001
		-	HV	TR5	29.3250	4.26	72.0	6.88642	< .001
		-	HV	TR6	30.0875	4.26	72.0	7.06548	< .001

Post Hoc Comparisons - Species ✻ Soil Mix

Comparison									
**Species**	**Soil Mix**		**Species**	**Soil Mix**	**Mean Difference**	**SE**	**df**	**t**	**p_{tukey}**
		-	TF	TR1	20.4000	4.26	72.0	4.79055	0.002
		-	TF	TR2	22.9500	4.26	72.0	5.38937	< .001
		-	TF	TR3	17.3250	4.26	72.0	4.06845	0.021
		-	TF	TR4	16.3000	4.26	72.0	3.82775	0.044
		-	TF	TR5	18.9750	4.26	72.0	4.45592	0.006
		-	TF	TR6	7.1875	4.26	72.0	1.68785	0.989
		-	SL	TR1	11.9750	4.26	72.0	2.81210	0.446
		-	SL	TR2	39.2250	4.26	72.0	9.21125	< .001
		-	SL	TR3	17.1000	4.26	72.0	4.01561	0.025
		-	SL	TR4	23.3875	4.26	72.0	5.49211	< .001
		-	SL	TR5	18.6875	4.26	72.0	4.38840	0.008
		-	SL	TR6	34.5375	4.26	72.0	8.11048	< .001
	TR5	-	SW	TR6	-6.1250	4.26	72.0	-1.43834	0.999
		-	HV	TR1	20.2875	4.26	72.0	4.76413	0.002
		-	HV	TR2	30.7625	4.26	72.0	7.22399	< .001
		-	HV	TR3	26.2550	4.26	72.0	6.16549	< .001
		-	HV	TR4	25.5750	4.26	72.0	6.00580	< .001
		-	HV	TR5	26.2000	4.26	72.0	6.15257	< .001
		-	HV	TR6	26.9625	4.26	72.0	6.33163	< .001
		-	TF	TR1	17.2750	4.26	72.0	4.05671	0.022
		-	TF	TR2	19.8250	4.26	72.0	4.65552	0.003
		-	TF	TR3	14.2000	4.26	72.0	3.33460	0.159
		-	TF	TR4	13.1750	4.26	72.0	3.09390	0.269
		-	TF	TR5	15.8500	4.26	72.0	3.72207	0.059
		-	TF	TR6	4.0625	4.26	72.0	0.95400	1.000
		-	SL	TR1	8.8500	4.26	72.0	2.07825	0.910
		-	SL	TR2	36.1000	4.26	72.0	8.47740	< .001
		-	SL	TR3	13.9750	4.26	72.0	3.28176	0.180
		-	SL	TR4	20.2625	4.26	72.0	4.75826	0.002
		-	SL	TR5	15.5625	4.26	72.0	3.65456	0.071

Post Hoc Comparisons - Species ✻ Soil Mix

Comparison									
**Species**	**Soil Mix**		**Species**	**Soil Mix**	**Mean Difference**	**SE**	**df**	**t**	**p_{tukey}**
		-	SL	TR6	31.4125	4.26	72.0	7.37663	< .001
	TR6	-	HV	TR1	26.4125	4.26	72.0	6.20247	< .001
		-	HV	TR2	36.8875	4.26	72.0	8.66233	< .001
		-	HV	TR3	32.3800	4.26	72.0	7.60383	< .001
		-	HV	TR4	31.7000	4.26	72.0	7.44414	< .001
		-	HV	TR5	32.3250	4.26	72.0	7.59091	< .001
		-	HV	TR6	33.0875	4.26	72.0	7.76997	< .001
		-	TF	TR1	23.4000	4.26	72.0	5.49505	< .001
		-	TF	TR2	25.9500	4.26	72.0	6.09386	< .001
		-	TF	TR3	20.3250	4.26	72.0	4.77294	0.002
		-	TF	TR4	19.3000	4.26	72.0	4.53224	0.005
		-	TF	TR5	21.9750	4.26	72.0	5.16041	< .001
		-	TF	TR6	10.1875	4.26	72.0	2.39234	0.746
		-	SL	TR1	14.9750	4.26	72.0	3.51659	0.102
		-	SL	TR2	42.2250	4.26	72.0	9.91574	< .001
		-	SL	TR3	20.1000	4.26	72.0	4.72010	0.002
		-	SL	TR4	26.3875	4.26	72.0	6.19660	< .001
		-	SL	TR5	21.6875	4.26	72.0	5.09290	< .001
		-	SL	TR6	37.5375	4.26	72.0	8.81497	< .001
HV	TR1	-	HV	TR2	10.4750	4.26	72.0	2.45985	0.700
		-	HV	TR3	5.9675	4.26	72.0	1.40135	0.999
		-	HV	TR4	5.2875	4.26	72.0	1.24167	1.000
		-	HV	TR5	5.9125	4.26	72.0	1.38844	0.999
		-	HV	TR6	6.6750	4.26	72.0	1.56750	0.996
		-	TF	TR1	-3.0125	4.26	72.0	-0.70743	1.000
		-	TF	TR2	-0.4625	4.26	72.0	-0.10861	1.000
		-	TF	TR3	-6.0875	4.26	72.0	-1.42953	0.999
		-	TF	TR4	-7.1125	4.26	72.0	-1.67024	0.991
		-	TF	TR5	-4.4375	4.26	72.0	-1.04206	1.000
		-	TF	TR6	-16.2250	4.26	72.0	-3.81013	0.046

Post Hoc Comparisons - Species ✻ Soil Mix

Comparison									
Species	Soil Mix		Species	Soil Mix	Mean Difference	SE	df	t	p_{tukey}
		-	SL	TR1	-11.4375	4.26	72.0	-2.68588	0.537
		-	SL	TR2	15.8125	4.26	72.0	3.71327	0.061
		-	SL	TR3	-6.3125	4.26	72.0	-1.48237	0.998
		-	SL	TR4	-0.0250	4.26	72.0	-0.00587	1.000
		-	SL	TR5	-4.7250	4.26	72.0	-1.10958	1.000
		-	SL	TR6	11.1250	4.26	72.0	2.61250	0.591
	TR2	-	HV	TR3	-4.5075	4.26	72.0	-1.05850	1.000
		-	HV	TR4	-5.1875	4.26	72.0	-1.21819	1.000
		-	HV	TR5	-4.5625	4.26	72.0	-1.07142	1.000
		-	HV	TR6	-3.8000	4.26	72.0	-0.89236	1.000
		-	TF	TR1	-13.4875	4.26	72.0	-3.16728	0.231
		-	TF	TR2	-10.9375	4.26	72.0	-2.56846	0.623
		-	TF	TR3	-16.5625	4.26	72.0	-3.88939	0.037
		-	TF	TR4	-17.5875	4.26	72.0	-4.13009	0.018
		-	TF	TR5	-14.9125	4.26	72.0	-3.50192	0.106
		-	TF	TR6	-26.7000	4.26	72.0	-6.26999	< .001
		-	SL	TR1	-21.9125	4.26	72.0	-5.14573	< .001
		-	SL	TR2	5.3375	4.26	72.0	1.25341	1.000
		-	SL	TR3	-16.7875	4.26	72.0	-3.94223	0.031
		-	SL	TR4	-10.5000	4.26	72.0	-2.46573	0.696
		-	SL	TR5	-15.2000	4.26	72.0	-3.56943	0.089
		-	SL	TR6	0.6500	4.26	72.0	0.15264	1.000
	TR3	-	HV	TR4	-0.6800	4.26	72.0	-0.15969	1.000
		-	HV	TR5	-0.0550	4.26	72.0	-0.01292	1.000
		-	HV	TR6	0.7075	4.26	72.0	0.16614	1.000
		-	TF	TR1	-8.9800	4.26	72.0	-2.10878	0.898
		-	TF	TR2	-6.4300	4.26	72.0	-1.50996	0.997
		-	TF	TR3	-12.0550	4.26	72.0	-2.83089	0.433
		-	TF	TR4	-13.0800	4.26	72.0	-3.07159	0.281
		-	TF	TR5	-10.4050	4.26	72.0	-2.44342	0.712

Post Hoc Comparisons - Species ✻ Soil Mix

Comparison									
**Species**	**Soil Mix**		**Species**	**Soil Mix**	**Mean Difference**	**SE**	**df**	**t**	p_{tukey}
		-	TF	TR6	-22.1925	4.26	72.0	-5.21149	< .001
		-	SL	TR1	-17.4050	4.26	72.0	-4.08723	0.020
		-	SL	TR2	9.8450	4.26	72.0	2.31191	0.796
		-	SL	TR3	-12.2800	4.26	72.0	-2.88372	0.397
		-	SL	TR4	-5.9925	4.26	72.0	-1.40722	0.999
		-	SL	TR5	-10.6925	4.26	72.0	-2.51093	0.664
		-	SL	TR6	5.1575	4.26	72.0	1.21114	1.000
	TR4	-	HV	TR5	0.6250	4.26	72.0	0.14677	1.000
		-	HV	TR6	1.3875	4.26	72.0	0.32583	1.000
		-	TF	TR1	-8.3000	4.26	72.0	-1.94910	0.950
		-	TF	TR2	-5.7500	4.26	72.0	-1.35028	0.999
		-	TF	TR3	-11.3750	4.26	72.0	-2.67120	0.547
		-	TF	TR4	-12.4000	4.26	72.0	-2.91190	0.378
		-	TF	TR5	-9.7250	4.26	72.0	-2.28373	0.812
		-	TF	TR6	-21.5125	4.26	72.0	-5.05180	< .001
		-	SL	TR1	-16.7250	4.26	72.0	-3.92755	0.033
		-	SL	TR2	10.5250	4.26	72.0	2.47160	0.692
		-	SL	TR3	-11.6000	4.26	72.0	-2.72404	0.509
		-	SL	TR4	-5.3125	4.26	72.0	-1.24754	1.000
		-	SL	TR5	-10.0125	4.26	72.0	-2.35125	0.772
		-	SL	TR6	5.8375	4.26	72.0	1.37083	0.999
	TR5	-	HV	TR6	0.7625	4.26	72.0	0.17906	1.000
		-	TF	TR1	-8.9250	4.26	72.0	-2.09587	0.903
		-	TF	TR2	-6.3750	4.26	72.0	-1.49705	0.998
		-	TF	TR3	-12.0000	4.26	72.0	-2.81797	0.442
		-	TF	TR4	-13.0250	4.26	72.0	-3.05867	0.288
		-	TF	TR5	-10.3500	4.26	72.0	-2.43050	0.720
		-	TF	TR6	-22.1375	4.26	72.0	-5.19857	< .001
		-	SL	TR1	-17.3500	4.26	72.0	-4.07432	0.021
		-	SL	TR2	9.9000	4.26	72.0	2.32483	0.788

Post Hoc Comparisons - Species ✻ Soil Mix

Comparison									
**Species**	**Soil Mix**		**Species**	**Soil Mix**	**Mean Difference**	**SE**	**df**	**t**	**p_{tukey}**
		-	SL	TR3	-12.2250	4.26	72.0	-2.87081	0.405
		-	SL	TR4	-5.9375	4.26	72.0	-1.39431	0.999
		-	SL	TR5	-10.6375	4.26	72.0	-2.49801	0.674
		-	SL	TR6	5.2125	4.26	72.0	1.22406	1.000
	TR6	-	TF	TR1	-9.6875	4.26	72.0	-2.27493	0.817
		-	TF	TR2	-7.1375	4.26	72.0	-1.67611	0.990
		-	TF	TR3	-12.7625	4.26	72.0	-2.99703	0.324
		-	TF	TR4	-13.7875	4.26	72.0	-3.23773	0.199
		-	TF	TR5	-11.1125	4.26	72.0	-2.60956	0.593
		-	TF	TR6	-22.9000	4.26	72.0	-5.37763	< .001
		-	SL	TR1	-18.1125	4.26	72.0	-4.25338	0.012
		-	SL	TR2	9.1375	4.26	72.0	2.14577	0.882
		-	SL	TR3	-12.9875	4.26	72.0	-3.04987	0.293
		-	SL	TR4	-6.7000	4.26	72.0	-1.57337	0.996
		-	SL	TR5	-11.4000	4.26	72.0	-2.67707	0.543
		-	SL	TR6	4.4500	4.26	72.0	1.04500	1.000
TF	TR1	-	TF	TR2	2.5500	4.26	72.0	0.59882	1.000
		-	TF	TR3	-3.0750	4.26	72.0	-0.72211	1.000
		-	TF	TR4	-4.1000	4.26	72.0	-0.96281	1.000
		-	TF	TR5	-1.4250	4.26	72.0	-0.33463	1.000
		-	TF	TR6	-13.2125	4.26	72.0	-3.10270	0.264
		-	SL	TR1	-8.4250	4.26	72.0	-1.97845	0.942
		-	SL	TR2	18.8250	4.26	72.0	4.42069	0.007
		-	SL	TR3	-3.3000	4.26	72.0	-0.77494	1.000
		-	SL	TR4	2.9875	4.26	72.0	0.70156	1.000
		-	SL	TR5	-1.7125	4.26	72.0	-0.40215	1.000
		-	SL	TR6	14.1375	4.26	72.0	3.31992	0.165
	TR2	-	TF	TR3	-5.6250	4.26	72.0	-1.32092	1.000
		-	TF	TR4	-6.6500	4.26	72.0	-1.56163	0.996
		-	TF	TR5	-3.9750	4.26	72.0	-0.93345	1.000

Post Hoc Comparisons - Species ✻ Soil Mix

Comparison									
**Species**	**Soil Mix**		**Species**	**Soil Mix**	**Mean Difference**	**SE**	**df**	**t**	p_{tukey}
		-	TF	TR6	-15.7625	4.26	72.0	-3.70152	0.063
		-	SL	TR1	-10.9750	4.26	72.0	-2.57727	0.616
		-	SL	TR2	16.2750	4.26	72.0	3.82187	0.045
		-	SL	TR3	-5.8500	4.26	72.0	-1.37376	0.999
		-	SL	TR4	0.4375	4.26	72.0	0.10274	1.000
		-	SL	TR5	-4.2625	4.26	72.0	-1.00097	1.000
		-	SL	TR6	11.5875	4.26	72.0	2.72110	0.511
	TR3	-	TF	TR4	-1.0250	4.26	72.0	-0.24070	1.000
		-	TF	TR5	1.6500	4.26	72.0	0.38747	1.000
		-	TF	TR6	-10.1375	4.26	72.0	-2.38060	0.753
		-	SL	TR1	-5.3500	4.26	72.0	-1.25635	1.000
		-	SL	TR2	21.9000	4.26	72.0	5.14280	< .001
		-	SL	TR3	-0.2250	4.26	72.0	-0.05284	1.000
		-	SL	TR4	6.0625	4.26	72.0	1.42366	0.999
		-	SL	TR5	1.3625	4.26	72.0	0.31996	1.000
		-	SL	TR6	17.2125	4.26	72.0	4.04203	0.023
	TR4	-	TF	TR5	2.6750	4.26	72.0	0.62817	1.000
		-	TF	TR6	-9.1125	4.26	72.0	-2.13990	0.885
		-	SL	TR1	-4.3250	4.26	72.0	-1.01564	1.000
		-	SL	TR2	22.9250	4.26	72.0	5.38350	< .001
		-	SL	TR3	0.8000	4.26	72.0	0.18786	1.000
		-	SL	TR4	7.0875	4.26	72.0	1.66436	0.991
		-	SL	TR5	2.3875	4.26	72.0	0.56066	1.000
		-	SL	TR6	18.2375	4.26	72.0	4.28273	0.011
	TR5	-	TF	TR6	-11.7875	4.26	72.0	-2.76807	0.477
		-	SL	TR1	-7.0000	4.26	72.0	-1.64382	0.992
		-	SL	TR2	20.2500	4.26	72.0	4.75533	0.002
		-	SL	TR3	-1.8750	4.26	72.0	-0.44031	1.000
		-	SL	TR4	4.4125	4.26	72.0	1.03619	1.000
		-	SL	TR5	-0.2875	4.26	72.0	-0.06751	1.000

Post Hoc Comparisons - Species ✻ Soil Mix

Comparison									
**Species**	**Soil Mix**		**Species**	**Soil Mix**	**Mean Difference**	**SE**	**df**	**t**	**p_{tukey}**
		-	SL	TR6	15.5625	4.26	72.0	3.65456	0.071
	TR6	-	SL	TR1	4.7875	4.26	72.0	1.12425	1.000
		-	SL	TR2	32.0375	4.26	72.0	7.52340	< .001
		-	SL	TR3	9.9125	4.26	72.0	2.32776	0.786
		-	SL	TR4	16.2000	4.26	72.0	3.80426	0.047
		-	SL	TR5	11.5000	4.26	72.0	2.70056	0.526
		-	SL	TR6	27.3500	4.26	72.0	6.42263	< .001
SL	TR1	-	SL	TR2	27.2500	4.26	72.0	6.39915	< .001
		-	SL	TR3	5.1250	4.26	72.0	1.20351	1.000
		-	SL	TR4	11.4125	4.26	72.0	2.68001	0.541
		-	SL	TR5	6.7125	4.26	72.0	1.57630	0.995
		-	SL	TR6	22.5625	4.26	72.0	5.29837	< .001
	TR2	-	SL	TR3	-22.1250	4.26	72.0	-5.19564	< .001
		-	SL	TR4	-15.8375	4.26	72.0	-3.71914	0.060
		-	SL	TR5	-20.5375	4.26	72.0	-4.82284	0.002
		-	SL	TR6	-4.6875	4.26	72.0	-1.10077	1.000
	TR3	-	SL	TR4	6.2875	4.26	72.0	1.47650	0.998
		-	SL	TR5	1.5875	4.26	72.0	0.37279	1.000
		-	SL	TR6	17.4375	4.26	72.0	4.09487	0.020
	TR4	-	SL	TR5	-4.7000	4.26	72.0	-1.10371	1.000
		-	SL	TR6	11.1500	4.26	72.0	2.61837	0.586
	TR5	-	SL	TR6	15.8500	4.26	72.0	3.72207	0.059

Note. Comparisons are based on estimated marginal means

Estimated Marginal Means
Soil Mix ✻ Species

Estimated Marginal Means - Soil Mix ✻ Species

				95% Confidence Interval	
**Species**	**Soil Mix**	**Mean**	**SE**	**Lower**	**Upper**
SW	TR1	35.39	3.01	29.385	41.39
	TR2	22.20	3.01	16.197	28.20
	TR3	27.11	3.01	21.110	33.12
	TR4	39.23	3.01	33.222	45.23
	TR5	36.10	3.01	30.097	42.10
	TR6	42.23	3.01	36.222	48.23
HV	TR1	15.81	3.01	9.810	21.82
	TR2	5.34	3.01	-0.665	11.34
	TR3	9.85	3.01	3.842	15.85
	TR4	10.53	3.01	4.522	16.53
	TR5	9.90	3.01	3.897	15.90
	TR6	9.14	3.01	3.135	15.14
TF	TR1	18.83	3.01	12.822	24.83
	TR2	16.28	3.01	10.272	22.28
	TR3	21.90	3.01	15.897	27.90
	TR4	22.93	3.01	16.922	28.93
	TR5	20.25	3.01	14.247	26.25
	TR6	32.04	3.01	26.035	38.04
SL	TR1	27.25	3.01	21.247	33.25
	TR2	2.15e-14	3.01	-6.003	6.00
	TR3	22.13	3.01	16.122	28.13
	TR4	15.84	3.01	9.835	21.84
	TR5	20.54	3.01	14.535	26.54
	TR6	4.69	3.01	-1.315	10.69

Estimated Marginal Means - Species ✻ Soil Mix

				95% Confidence Interval	
**Soil Mix**	**Species**	**Mean**	**SE**	**Lower**	**Upper**
TR1	SW	35.39	3.01	29.385	41.39
	HV	15.81	3.01	9.810	21.82
	TF	18.83	3.01	12.822	24.83
	SL	27.25	3.01	21.247	33.25
TR2	SW	22.20	3.01	16.197	28.20
	HV	5.34	3.01	-0.665	11.34
	TF	16.28	3.01	10.272	22.28
	SL	2.15e-14	3.01	-6.003	6.00
TR3	SW	27.11	3.01	21.110	33.12
	HV	9.85	3.01	3.842	15.85
	TF	21.90	3.01	15.897	27.90
	SL	22.13	3.01	16.122	28.13
TR4	SW	39.23	3.01	33.222	45.23
	HV	10.53	3.01	4.522	16.53
	TF	22.93	3.01	16.922	28.93
	SL	15.84	3.01	9.835	21.84
TR5	SW	36.10	3.01	30.097	42.10
	HV	9.90	3.01	3.897	15.90
	TF	20.25	3.01	14.247	26.25
	SL	20.54	3.01	14.535	26.54
TR6	SW	42.23	3.01	36.222	48.23
	HV	9.14	3.01	3.135	15.14
	TF	32.04	3.01	26.035	38.04
	SL	4.69	3.01	-1.315	10.69

Species

Estimated Marginal Means - Species

			95% Confidence Interval	
**Species**	**Mean**	**SE**	**Lower**	**Upper**
SW	33.7	1.23	31.26	36.2
HV	10.1	1.23	7.64	12.5
TF	22.0	1.23	19.58	24.5
SL	15.1	1.23	12.62	17.5

Soil Mix

Estimated Marginal Means - Soil Mix

			95% Confidence Interval	
**Soil Mix**	**Mean**	**SE**	**Lower**	**Upper**
TR1	2<br>4.3	1.51	21.32	27.3
TR2	11.0	1.51	7.95	14.0
TR3	20.2	1.51	17.24	23.2
TR4	22.1	1.51	19.13	25.1
TR5	21.7	1.51	18.70	24.7
TR6	22.0	1.51	19.02	25.0

2. Root weights

ANOVA - Root_wt

	Sum of Squares	df	Mean Square	F	p
Species	8251	3	2750.4	20.845	< .001
Soil Mix	890	5	177.9	1.349	0.254
Species ✻ Soil Mix	1329	15	88.6	0.671	0.804
Residuals	9500	72	131.9		

Post Hoc Tests

Post Hoc Comparisons - Species

Comparison							
**Species**		**Species**	**Mean Difference**	**SE**	**df**	**t**	**p_{tukey}**
SW	-	HV	23.76	3.32	72.0	7.164	< .001
	-	TF	12.22	3.32	72.0	3.684	0.002
	-	SL	21.05	3.32	72.0	6.348	< .001
HV	-	TF	-11.54	3.32	72.0	-3.480	0.005
	-	SL	-2.71	3.32	72.0	-0.816	0.847
TF	-	SL	8.83	3.32	72.0	2.664	0.046

Note. Comparisons are based on estimated marginal means

Estimated Marginal Means
Species

Estimated Marginal Means - Species

			95% Confidence Interval	
**Species**	**Mean**	**SE**	**Lower**	**Upper**
SW	27.45	2.34	22.774	32.12
HV	3.69	2.34	-0.982	8.37
TF	15.23	2.34	10.557	19.91
SL	6.40	2.34	1.724	11.07

Soil Mix

Estimated Marginal Means - Soil Mix

			95% Confidence Interval	
**Soil Mix**	**Mean**	**SE**	**Lower**	**Upper**
TR1	12.09	2.87	6.37	17.8
TR2	8.45	2.87	2.73	14.2
TR3	15.46	2.87	9.73	21.2
TR4	13.79	2.87	8.06	19.5
TR5	18.00	2.87	12.28	23.7
sTR6	11.36	2.87	5.64	17.1
